# Growth Modeling in a Diagnostic Classification Model (DCM) Framework–A Multivariate Longitudinal Diagnostic Classification Model

**DOI:** 10.3389/fpsyg.2020.01714

**Published:** 2020-08-07

**Authors:** Qianqian Pan, Lu Qin, Neal Kingston

**Affiliations:** ^1^Department of Educational Psychology, The University of Kansas, Lawrence, KS, United States; ^2^Institutional Research and Assessment, Howard University, Washington, DC, United States

**Keywords:** diagnostic classification model, longitudinal data analysis, growth model, cognitive diagnostic assessment, multivariate

## Abstract

A multivariate longitudinal DCM is developed that is the composite of two components, the log-linear cognitive diagnostic model (LCDM) as the measurement model component that evaluates the mastery status of attributes at each measurement occasion, and a generalized multivariate growth curve model that describes the growth of each attribute over time. The proposed model represents an improvement in the current longitudinal DCMs given its ability to incorporate both balanced and unbalanced data and to measure the growth of a single attribute directly without assuming that attributes grow in the same pattern. One simulation study was conducted to evaluate the proposed model in terms of the convergence rates, the accuracy of classification, and parameter recoveries under different combinations of four design factors: the sample size, the growth patterns, the G matrix design, and the number of measurement occasions. The results revealed the following: (1) In general, the proposed model provided good convergence rates under different conditions. (2) Regarding the classification accuracy, the proposed model achieved good recoveries on the probabilities of attribute mastery. However, the correct classification rates depended on the cut point that was used to classify individuals. For individuals who truly mastered the attributes, the correct classification rates increased as the measurement occasions increased; however, for individuals who truly did not master the attributes, the correct classification rates decreased slightly as the numbers of measurement occasions increased. Cohen's kappa increased as the number of measurement occasions increased. (3) Both the intercept and main effect parameters in the LCDM were recovered well. The interaction effect parameters had a relatively large bias under the condition with a small sample size and fewer measurement occasions; however, the recoveries were improved as the sample size and the number of measurement occasions increased. (4) Overall, the proposed model achieved acceptable recoveries on both the fixed and random effects in the generalized growth curve model.

## Introduction

Diagnostic classification models (DCMs; e.g., Rupp et al., 2010), also referred to as cognitive diagnosis models (CDMs; e.g., Leighton and Gierl, 2007), are defined as a family of confirmatory multidimensional latent-variable models with categorical latent variables (Rupp et al., 2010). DCMs evaluate the student's mastery status on each latent variable from a set of narrowly defined latent variables, referred to attributes in the DCM literature, and then classify students into attribute profiles that were determined as a priori (DiBello et al., 1995). DCMs provide fine-grained and multidimensional diagnostic information, which could help educators adjust classroom instruction and improve student learning. Since the traditional scale scores (e.g., IRT scores) have limits in providing enough information to inform classroom instruction and learning (e.g., de La Torre, 2009), DCMs have received growing attention in the educational measurement community as well as from educational practitioners in recent years.

DCMs have been increasingly used for empirical data analysis in recent years. For example, DCMs have been retrofitted to existing large-scale assessments to identify examinees' mastery status of tested skills (e.g., Lee and Sawaki, 2009; George and Robitzsch, 2014; Sedat and Arican, 2015; Ravand, 2016). In addition, some researchers successfully demonstrated the practical uses of DCMs in test development (Bradshaw et al., 2014). DCMs have also been applied in one large-scale assessment program (Dynamic Learning Maps® alternate assessment; DLM® Dynamic Learning Maps, 2016) to detect distinct patterns of skill mastery for students with significant cognitive disabilities. However, most applications of DCMs are static, meaning that DCMs are used to classify individuals at a single time point. When longitudinal data are modeled, the longitudinal DCM is used to measure the change in the attribute profiles and mastery status over time.

Currently, two types of longitudinal DCMs have been proposed to analyze longitudinal data in the DCM framework. Latent transition analysis (LTA; Collins and Wugalter, 1992)—based longitudinal DCMs (e.g., Li et al., 2016; Kaya and Leite, 2017; Madison and Bradshaw, 2018) estimate the probabilities of transitioning from one latent class to another latent class or staying at the same latent class across two measurement occasions. Higher-order DCM (HDCM; e.g., de la Torre and Douglas, 2004; Templin and Bradshaw, 2014)—based longitudinal DCMs (e.g., Huang, 2017; Zhan et al., 2019) assumes a higher-order continuous factor to predict the mastery status of lower-order attributes so that the changes in the higher-order factor are used to infer the changes of lower-order attributes over time.

These two longitudinal DCM approaches have been evaluated by a few simulation studies and some applied research, which has demonstrated their utility for analyzing longitudinal data in the DCM framework. However, these models are not without limitations. For example, LTA-based longitudinal DCMs are restricted to the balanced data^1^ and assume attributes are independent. In addition, LTA-based approach is limited to assessing changes between only two measurement occasions (Huang, 2017). On the other hand, HDCM-based longitudinal DCMs assume all attributes have similar growth trajectories. However, previous studies found attributes could change in different ways (e.g., Li et al., 2016; Madison and Bradshaw, 2018).

So, the overarching goal of the current study is to develop a multivariate longitudinal DCM, improves upon current longitudinal DCMs by (1) being able to incorporate both balanced data and unbalanced data and (2) measuring the growth of multiple attributes that have dissimilar growth trajectories. More specific research questions are presented in the Research Design and Methods section.

## Longitudinal Diagnostic Classification Models

Currently, two types of longitudinal DCMs have been developed and applied to measure longitudinal data, including latent transition analysis (LTA; Collins and Wugalter, 1992)-based longitudinal DCMs (e.g., Li et al., 2016; Kaya and Leite, 2017; Madison and Bradshaw, 2018), and Higher-order DCM (HDCM; e.g., de la Torre and Douglas, 2004; Templin and Bradshaw, 2014)—based longitudinal DCMs (e.g., Huang, 2017; Zhan et al., 2019). The definitions, model specifications, and limitations of these two types of longitudinal DCMs are briefly reviewed as follows.

### LTA-Based Longitudinal DCMs

Latent class analysis (LCA; e.g., Lazarsfeld and Henry, 1968; Goodman, 1974) is developed for analyzing categorical latent variables. Latent transition analysis (LTA) is the extension of the general LCA for longitudinal data, which enables the estimation of both the latent class membership probability, often called the latent status prevalence in the LTA, and the probabilities of transitions in latent status from one measurement occasion to the next (Lanza et al., 2003, p. 161). LTA-based longitudinal DCMs are a composite of the DCM, as the measurement model to classify individuals into different latent classes at each time point, and the LTA, as the structural model to estimate the transition probability to represent the changes in latent class membership across two measurement occasions.

A few LTA-based longitudinal DCMs have been evaluated in simulation studies as well as applied in empirical studies. For example, Li et al. (2016) used the LTA with DINA(the deterministic-input, noisy-and-gate model; Junker and Sijtsma, 2001) as the measurement model to evaluate the effectiveness of an intervention for four cognitive skills across four measurement occasions for a sample of 109 seventh-grade students. This study provided base-rates of cognitive skills at each measurement occasion and three conditional transition probabilities from Occasion 1 to Occasion 2, Occasion 2 to Occasion 3, and Occasion 3 to Occasion 4, respectively. The results showed that attributes had different base-rates at the beginning and different conditional transition probabilities over time.

Madison and Bradshaw (2018) proposed the transitional diagnostic classification model (TDCM) to measure growth in attribute mastery for pre-test and post-test data, where the LCDM was adopted as the measurement model along with the LTA as the structural model. A simulation study showed that the TDCM could provide accurate and reliable classification and transition probabilities overtime under the variations in the number of attributes, sample size, Q-matrix, pre-test, and post-test base-rates, and marginal mastery transition probabilities. Additionally, the TDCM was applied to two empirical studies. In both studies, four mathematic skills were assessed before and after an intervention. The results showed that the base-rates of all attributes were improved after the intervention. However, the improvement differed by attributes and the groups, e.g., the control group or the intervention groups.

Furthermore, Chen et al. (2018) proposed a family of first-order hidden Markov models (FOHM) to model the learning trajectories with the CDM framework. Compared to the aforementioned LTA-based longitudinal DCMs that estimated the transition probabilities between two measurement occasions, FOHMs could estimate a transition probability matrix across multiple measurement occasions, which shows the probabilities of remaining in the same latent stage or learning some attributes or even losing some attributes from time *t* to *t* + 1. Such that it could provide an entire learning trajectory across time. Also, Chen et al. (2018) emphasized that there might be different types of learning trajectories, including the unstructured trajectories and non-decreasing trajectories. And, FOHMs are very flexible to estimate not only the most general trajectories but also some more parsimonious trajectories. So, even though the number of parameters in the transition probability matrix increases exponentially with the number of measurement occasions increasing, the restricted learning patterns could reduce the number of parameters.

### Higher-Order DCM-Based Longitudinal DCMs

Higher-order DCMs (HDCMs) parameterize the structural model of general DCMs in a certain way to reduce the numbers of structural parameters. Several approaches have been utilized to construct the structural model (e.g., Hartz, 2002; de la Torre and Douglas, 2004; Rupp and Templin, 2008). The majority of HDCM-based longitudinal DCMs are parameterized using the logistic regression models (e.g., Huang, 2017; Zhan et al., 2019), which are composites of two model components. The first component is the HDCM, where a higher-order continuous factor, θ_*rt*_, is assumed to predict the mastery statuses of multiple lower-order attributes at time *t*. The second component is the univariate growth curve models (GCMs; e.g., Raghavarao and Padgett, 2014; Hoffman, 2015), which describes the inter- and intra-individual differences in changes of this higher-order factor over *T* time points.

Recently, Huang (2017) proposed an HDCM-based longitudinal DCM, where a G-DINA model was used to evaluate the mastery status of attributes at each time point. Then, the Rasch model was utilized to construct the higher-order DCM at each time point. Last, a univariate GCM was applied to describe the growth of the higher-order factor over time. In addition, a set of time-invariant predictors (e.g., gender, age) were included to predict the random intercept and slope. This HDCM-based longitudinal DCM was evaluated in three simulation studies which varied several factors, including the sample size, the test length, the number of attributes, the item difficulty, and the number of measurement occasions. The results showed that a large sample size (1,000 individuals), enough items (30 items), and more measurement occasions (3 measurement occasions) could improve the parameter recovery and classification accuracy. Additionally, this HCDM-based longitudinal DCM was retrofitted to an empirical testing data, which assessed four attributes in a group of 4,177 high school students across three measurement occasions. The results showed that attributes differed in both the initial base-rates and the amount of improvement of the base-rates, for example, the base-rates of the “geometry” attribute were 0.90, 0.89, and 0.92 across three measurement occasions; however, the base-rates of the “number” attribute were 0.36, 0.49, and 0.58 across three measurement occasions. These results indicated different attributes developed different growth rates. Also, Zhan et al. (2019) developed a Long-DINA model, where (1) a DINA model was used to determine the mastery status of attributes at each time point, (2) the examinee's general ability at each measurement occasion was predicted by mastery status of attributes through a 2PL multidimensional higher-order latent structural model, and (3) the mean differences between the general abilities estimated from different measurement occasions represented the growth of examinees. Furthermore, the main improvement of this model was that incorporated specific factors in the DINA model to capture local item dependence due to the repeated measure rather than assuming the measurement invariance across time.

### Limitations of Current Longitudinal DCMs

Even though the current longitudinal DCMs have provided a few approaches to analyze longitudinal data in the DCM framework; these longitudinal DCMs have limitations that could restrict their usage with empirical data. As discussed above, LTA-based longitudinal DCMs could estimate the changes of attributes directly over time. However, this method required balanced data. In other words, the time interval between measurement occasions cannot be accounted for in the model. This might result in inaccurately estimated transition probabilities if examinees have a different time interval between administrations. On the other hand, HDCM-based longitudinal DCMs estimate the growth of the higher-order factor via the univariate GCM framework, which could cooperate both balanced and unbalanced data. However, HDCM-based longitudinal models measure the growth of higher-order factors to indicate the growth of lower-order attributes, indicating multiple attributes should have similar growth patterns. While empirical studies' demonstrated attributes had different growth patterns, some attributes were improved over time, and some attributes had a nearly consistent base-rate over time. For example, Madison and Bradshaw (2018) measured the changes in mastery status for four mathematics skills using pre- and post-test data and found the base-rate of one attribute was almost constant, where the base-rates changed from 0.65 to 0.70. However, base-rates of another three attributes improved more, ranging from 0.38 to 0.58, 0.38 to 0.51, and 0.59 to 0.73, respectively. Therefore, it is not reasonable to assume all attributes have the same growth patterns such that the growth of the higher-order factor cannot represent the changes in lower-order attributes well.

Therefore, there is a need to improve the current longitudinal DCMs. The motivation for the current study is to improve the current longitudinal DCMs by developing a multivariate longitudinal DCM, which could incorporate both balanced and unbalanced data, and measure the growth of attributes directly without assuming that attributes have similar growth patterns.

## Research Design and Method

### Multivariate Longitudinal Diagnostic Classification Models

The proposed multivariate longitudinal DCM is a composite of two components, the LCDM as the measurement model component that evaluates the mastery status of attributes at each measurement occasion, and a generalized multivariate growth curve model (e.g., GCM; MacCallum et al., 1997; Goldstein, 2011; Hoffman, 2015) as the structural model component that describes the changes of attributes over time via a logistic link function.

#### Model Specification

Let *x*_*i*_ denote the item response of item *i*. Only the binary item response was considered in the current study; however, polytomous item responses could be incorporated as well. Let t = 1, 2, …, T denotes the number of measurement occasions; k = 1, 2, …, K denote the number of attributes; and $\alpha_{\text{rt}}^{\text{k}}=\alpha_{\text{rt}}^{1},\;\alpha_{\text{rt},\;\ldots ,\;}^{2}\alpha_{\text{rt}}^{\text{k}}$ denote the attribute profile at time *t*.

A three-level model is considered in the current study; Level 1 is the item level, Level 2 was the within-person level, and Level 3 is the between-person level.

In Level 1, the LCDM estimates the probability of individual *r* answering item *i* correct given profile α_r_ at time t, as shown in Equation (1), where λ_*i*, 0_ is the intercept parameter of the LCDM, indicating the logit of guessing the item *i* correctly without mastering any attributes, $\lambda_{i}^{T}$ is a vector of size (2^*K*^−1) × 1 with main effect and interaction parameters for item *i* at Time T, *q*_*i*_ is the set of Q matrix entries for item *i*, and *h* (α_*rt*_, *q*_*i*_) is a vector of size (2^*K*^−1) × 1 with linear combinations of the α_*rt*_ and *q*_*i*_.

For example, as shown in Table 2, the item 4 measures both Attribute 1 and Attribute 2 across all measurement occasions, such that, Equation (1) expresses the probability of a correct response to Item 4 is a function of the intercept (λ_1, 0_), the simple main effects of attribute 1 (λ_1, 1, (1)_) and attribute 2 (λ_1, 1, (2)_), interaction effects between these two attributes (λ_1, 2, (1, 2)_), and the mastery status of two attributes. The intercept represents the log-odds of a correct answer for individuals who did not master any of the attributes. The simple main effects of attributes represent the increase in log-odds for individuals who have mastered only one of the attributes. Moreover, the interaction represents the change in log-odds for individuals who have mastered both attributes. Since the attributes are all dichotomous, α_1_ = 1 indicates attribute 1 is mastered, while α_1_ = 0 indicates attribute 1 is not mastered. As mentioned, as a general diagnostic model, the LCDM is able to subsume other frequently used DCMs. Using the same example, when two main effects are fixed to 0, the DINA model is achieved (Bradshaw and Madison, 2016).

(1) $$\begin{array}{l} P\left(X_{4}=1|\alpha_{c}\right)=\\ \;\frac{\exp\left(\lambda_{1,0}+\lambda_{1,1,\left(1\right)}\left(\alpha_{1}\right)+\lambda_{1,1,\left(2\right)}\left(\alpha_{2}\right)+\lambda_{1,2,\left(1,2\right)}\left(\alpha_{1}\cdot \alpha_{2}\right)\right)}{1+\exp\left(\lambda_{1,0}+\lambda_{1,1,\left(1\right)}\left(\alpha_{1}\right)+\lambda_{1,1,\left(2\right)}\left(\alpha_{2}\right)+\lambda_{1,2,\left(1,2\right)}\left(\alpha_{1}\cdot \alpha_{2}\right)\right)} \end{array}$$

In Level 2, $\alpha_{\text{rt}}^{\text{k}}$ represents the mastery status of attribute k at time t, Time_rt_ represents the time variable for individual i at time t. Then, the log-odds of $P\left(\alpha_{\text{rt}}^{\text{k}}=1\right)$, indicating the probability of mastering attribute *k* at time *t*, are predicted by the random intercept $\beta_{\text{r}0}^{\text{k}}$ and random slope $\beta_{\text{r}1}^{\text{k}}$.

In Level 3, the random intercept $\beta_{0\text{r}}^{\text{k}}$ and random slope $\beta_{1\text{r}}^{\text{k}}$ are predicted by the average initial level $\gamma_{00}^{\text{k}}$ and average slope $\gamma_{10}^{\text{k}}$, respectively. ${\text{u}}_{0r}^{k}$ and ${\text{u}}_{1r}^{k}$ represent the individual r's deviations from the average initial level and growth rate for attribute k.

(2) $$\begin{array}{rcl} \text{Level }1\;\pi_{irt}=P\left(X_{irt}=1|\alpha_{rt}\right) & = & \frac{\exp\left(\lambda_{i,0}+\lambda_{i}^{T}h\left(\alpha_{rt},q_{i}\right)\right)}{1+\exp\left(\lambda_{i,0}+\lambda_{i}^{T}h\left(\alpha_{rt},q_{i}\right)\right)} \end{array}$$

(3) $$\begin{array}{lll} \text{Level }2\;logit\left(P\left(\alpha_{rt}^{k}=1\right)\right) & = & \beta_{r0}^{k}+\beta_{r1}^{k}\;Time_{rt}+\epsilon_{rt}^{k} \end{array}$$

(4) $$\begin{array}{lll} R & = & \left[\begin{array}{ccccc} \frac{\pi^{2}}{3} & \; & \cdots & \; & \;\\ 0 & \frac{\pi^{2}}{3} & \ldots & \; & \;\\ \vdots & \vdots & \ddots & \vdots & \vdots \\ 0 & 0 & \cdots & \frac{\pi^{2}}{3}\\ 0 & 0 & \ldots & 0 & \frac{\pi^{2}}{3} \end{array}\right] \end{array}$$

(5) $$\begin{array}{rcl} \beta_{0r}^{k} & = & \gamma_{00}^{k}+u_{0r}^{k} \end{array}$$

(6) $$\begin{array}{rcl} \beta_{1r}^{k} & = & \gamma_{10}^{k}+u_{1r}^{k} \end{array}$$

(7) $$\begin{array}{r} \text{Level }3\;\left[\begin{array}{cccc} \sigma_{u_{0}}^{\left(1\right)2}\\ \sigma_{u_{0}^{1},u_{1}^{1}} & \sigma_{u_{1}}^{\left(1\right)2}\\ \vdots & \ddots \\ \sigma_{u_{0}^{1},u_{0}^{K}} & \sigma_{u_{1}^{1},u_{0}^{K}} & \sigma_{u_{0}}^{\left(K\right)2}\\ \sigma_{u_{0}^{1},u_{1}^{K}} & \sigma_{u_{1}^{1},u_{1}^{K}} & \sigma_{u_{0}^{K},u_{1}^{K}} & \sigma_{u_{1}}^{\left(K\right)2} \end{array}\right] \end{array}$$

As shown in Equation (3), $\epsilon_{\text{rt}}^{\text{k}}$ are the Level 2 residuals, which follow a multivariate normal distribution with means of 0 and TK × TK covariance matrix of *R*, the diagonal elements are $\frac{\pi^{2}}{3}$, and off-diagonal elements are fixed to 0, indicating there are no covariances among **ϵ**_**rt**_ across constructs. In Level 3 variance $\left[u_{0r}^{k},\;u_{1r}^{k}]\;\sim \;MVN(0,\;G\right)$, G is a *KP* × *KP* covariance matrix, and *P* is the number of Level 2 random effects (Pan, 2018).

#### Research Questions

The purpose of the current study is to develop a multivariate longitudinal DCM and evaluate it under several conditions.

This study aims to answer the following research questions:

1. Does the proposed model provide satisfied classification accuracy under different conditions?
2. Do the sample size, the growth patterns, and the number of measurement occasions, the G matrix design, and their interactions impact the item parameter recoveries in the measurement model?
3. Do the sample size, the growth patterns, and the number of measurement occasions, the G matrix design, and their interactions impact the fixed and random effects recoveries in the generalized growth curve model?

### Simulation Design

To answer three research questions listed above, a simulation study was conducted, which included four design factors, (1) the sample size; (2) the growth patterns across attributes; (3) the G matrix design; and (4) the number of measurement occasions. Factors including the Q-matrix, the test length, the initial base-rate, and the item parameters were fixed. Simulation conditions are described below.

#### Design Factors

##### Sample size

The current study varied the sample size by 100, 200, and 300 to investigate the requirement for the sample size in the proposed model. Previous simulation studies in longitudinal DCMs used to have a large sample size that normally ranged from 500 to 3,000 (e.g., Kaya and Leite, 2017; Zhan et al., 2019; Madison and Bradshaw, 2018). However, the empirical studies usually had a relatively smaller sample size, normally ranging from 100 to 400 (e.g., Li et al., 2016). Therefore, it was useful to investigate the sufficient sample size for the proposed model to detect the growth of attributes over time, which could guide applied researchers to collect adequate participants without a waste of time and money.

##### Growth patterns across attributes

The proposed multivariate longitudinal DCM improves the current HDCM-based longitudinal DCMs in its potential for estimating the growth of attributes without assuming that attributes have similar growth trajectories. To examine if the proposed model could measure attributes with different growth patterns and attributes with similar growth patterns equally well, two different growth patterns across attributes were considered in the current study: (1) the even growth pattern in which attributes had similar growth patterns over time and (2) the uneven growth pattern in which attributes had different growth patterns over time.

**Figure 1:**
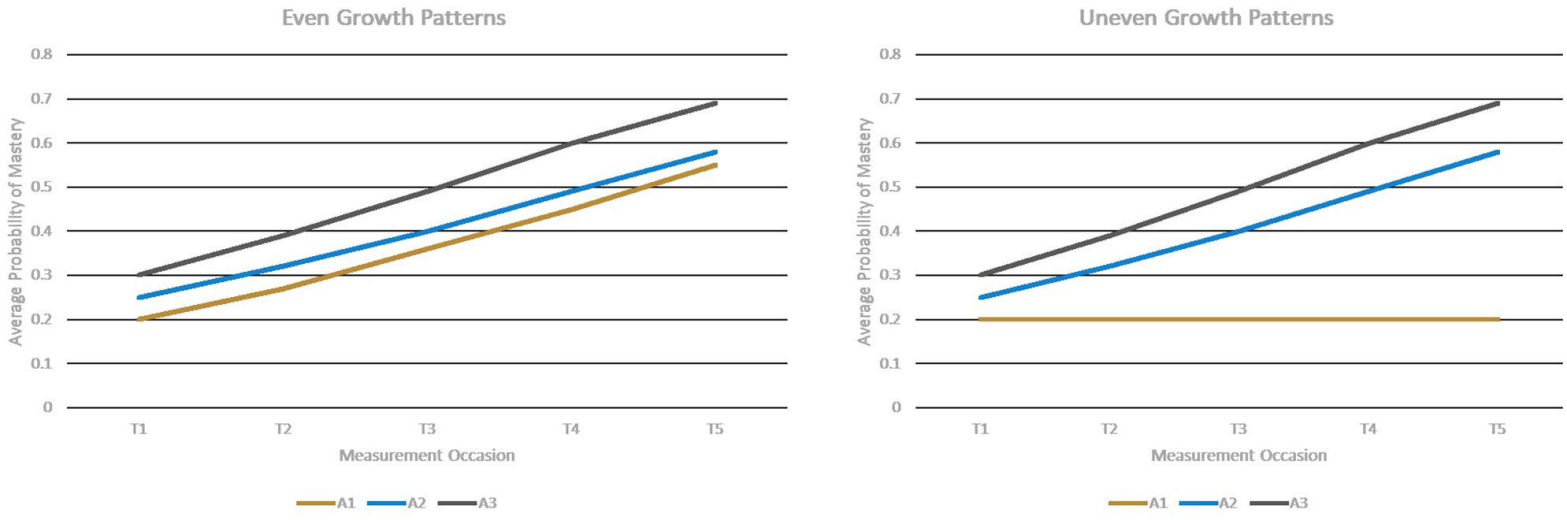
Two patterns of growth across attributes.

Figure 1 describes these two conditions, where *T*1–*T*5 represent the first to the fifth measurement occasion; *A*1, *A*2, and *A*3 represent Attribute 1, Attribute 2, and Attribute 3, respectively.

Under the even growth pattern condition, the base-rates of all three attributes were improved from the first measurement occasion to the last measurement occasion. Under the uneven growth pattern condition, the base-rates of Attributes 2 and 3 were improved across five measurement occasions, but the base-rates of Attribute 1 kept constant over time.

##### G matrix design

The G matrix plays an important role in the multivariate GCM, which reflects the relationships between outcomes across time. It is one of the main interests in the longitudinal studies that measure multiple outcomes over time (e.g., Hoffman, 2015).

To examine if the proposed multivariate longitudinal DCM can detect the relationships among attributes, two types of G matrices are considered in the current study: (1) under the equal correlation condition, all attributes had equal correlations between intercept, slopes, and intercept and slope, meaning that attributes are equally correlated, and (2) under the unequal correlation condition, as described in Figure 1, Attribute 2 and Attribute 3 had equal correlations between intercept, slopes, and intercept and slope, but Attribute 1 had lower correlations with Attribute 2 and 3. Table 1 presents the two types of G matrices and corresponding correlation matrices.

**Table 1:** G matrix specification and corresponding correlation matrix.

	**Equal correlation condition**						**Unequal correlation condition**					
	**$u_{0}^{1}$**	**$u_{1}^{1}$**	**$u_{0}^{2}$**	**$u_{1}^{2}$**	**$u_{0}^{3}$**	**$u_{1}^{3}$**	**$u_{0}^{1}$**	**$u_{1}^{1}$**	**$u_{0}^{2}$**	**$u_{1}^{2}$**	**$u_{0}^{3}$**	**$u_{1}^{3}$**
**CORRELATION MATRIX**												
$u_{0}^{1}$	**1.0**						**1.0**					
$u_{1}^{1}$	0.20	**1.0**					0.10	**1.0**				
$u_{0}^{2}$	0.90	0.10	**1.0**				0.90	0.01	**1.0**			
$u_{1}^{2}$	0.10	0.25	0.20	**1.0**			0.01	0.01	0.20	**1.0**		
$u_{0}^{3}$	0.90	0.10	0.90	0.10	**1.0**		0.10	0.01	0.90	0.10	**1.0**	
$u_{1}^{3}$	0.10	0.25	0.10	0.25	0.20	**1.0**	0.01	0.01	0.10	0.25	0.20	**1.0**
**COVARIANCE MATRIX**												
	$\sigma_{u_{0}^{1}}^{2}$	$\sigma_{u_{1}^{1}}^{2}$	$\sigma_{u_{0}^{2}}^{2}$	$\sigma_{u_{1}^{2}}^{2}$	$\sigma_{u_{0}^{3}}^{2}$	$\sigma_{u_{1}^{3}}^{2}$	$\sigma_{u_{0}^{1}}^{2}$	$\sigma_{u_{1}^{1}}^{2}$	$\sigma_{u_{0}^{2}}^{2}$	$\sigma_{u_{1}^{2}}^{2}$	$\sigma_{u_{0}^{3}}^{2}$	$\sigma_{u_{1}^{3}}^{2}$
$\sigma_{u_{0}^{1}}^{2}$	**0.1500**						**0.1500**					
$\sigma_{u_{1}^{1}}^{2}$	0.0173	**0.0500**					0.0173	**0.0500**				
$\sigma_{u_{0}^{2}}^{2}$	0.1350	0.0087	**0.1500**				0.1350	0.0009	**0.1500**			
$\sigma_{u_{1}^{2}}^{2}$	0.0087	0.0125	0.0173	**0.0500**			0.0009	0.0005	0.0173	**0.0500**		
$\sigma_{u_{0}^{3}}^{2}$	0.1350	0.0087	0.1350	0.0087	**0.1500**		0.1350	0.0009	0.1350	0.0087	**0.1500**	
$\sigma_{u_{1}^{3}}^{2}$	0.0087	0.0125	0.0087	0.0125	0.0173	**0.0500**	0.0009	0.0005	0.0087	0.0125	0.0173	**0.0500**

*$u_{0}^{k}$ and $u_{1}^{k}$ represent the random intercept and slope for attributes; $\sigma_{u_{0}^{k}}^{2}$ and $\sigma_{u_{0}^{k}}^{2}$ represent the random intercept and slope variance for attributes. Bold values means the correlation of this parameter itself*.

##### Number of measurement occasions

Previous simulation studies in HDCM-based longitudinal DCMs showed inconsistent results in the impacts of the number of measurement occasions on the classification accuracy. Huang (2017) found the number of measurement occasions (e.g., 2 or 3 measurement occasions) did not influence the classification accuracy significantly. However, Zhan et al. (2019) found the classification accuracy slightly increased as the number of measurement occasions increased. For the growth model, more measurement occasions are associated with good parameter recoveries (e.g., Preacher et al., 2008). To examine whether the number of measurement occasions impacted the performance of the proposed multivariate longitudinal DCM, the number of measurement occasions varied between 3 and 5 in the current study.

#### Fixed Conditions

##### Test length

A test of 30 binary items was simulated in the current study. The test length fell within the range of applied research as well as simulation studies in the longitudinal DCMs (e.g., Huang, 2017; Kaya and Leite, 2017; Madison and Bradshaw, 2018).

##### Q-matrix

As discussed above, DCMs are able to incorporate both the simple structure and the complex structure of the Q-matrix. In the current study, a complex structure of the Q-matrix was specified as shown in Table 2. Each item measures up to two attributes and attributes were assessed by equal numbers of items. This Q-matrix design was suggested by previous applied research and simulation studies (e.g., Bradshaw and Templin, 2014; Bradshaw et al., 2014; Kaya and Leite, 2017; Madison and Bradshaw, 2018).

**Table 2:** Q-matrix design.

**Item**	**Attribute 1**	**Attribute 2**	**Attribute 3**	**Item**	**Attribute 1**	**Attribute 2**	**Attribute 3**
1	1	0	0	16	1	1	0
2	0	1	0	17	1	0	1
3	0	0	1	18	0	1	1
4	1	1	0	19	1	0	0
5	1	0	1	20	0	1	0
6	0	1	1	21	0	0	1
7	1	0	0	22	1	1	0
8	0	1	0	23	1	0	1
9	0	0	1	24	0	1	1
10	1	1	0	25	1	0	0
11	1	0	1	26	0	1	0
12	0	1	1	27	0	0	1
13	1	0	0	28	1	1	0
14	0	1	0	29	1	0	1
15	0	0	1	30	0	1	1

##### Initial base-rates

The initial base-rate was fixed to 0.20, 0.25, and 0.30 for Attribute 1, Attribute 2, and Attribute 3, respectively. The previous empirical studies on measuring growth of attributes found initial base-rates ranged from 0.02 to 0.90 and suggested an easier attribute might have a base-rate approximately 0.60, a medium attribute might have a base-rate approximately 0.40, and a hard attribute might have a base-rate ~0.20 (Madison and Bradshaw, 2018); therefore, the base-rates are set to 0.20, 0.25, and 0.30 to mimic the hard, medium-hard, and medium attributes at the first measurement occasion.

##### Fixed effects ($\gamma_{00}^{\text{k}},\;\gamma_{01}^{\text{k}}$)

The linear growth of the log-odds of the probability of mastering attributes was considered in the current study. It should be noted that the linear growth of the log-odds of the probability did not necessarily result in the linear growth of base-rates over time. Table 3 presents the fixed effects under both even and uneven growth pattern conditions.

**Table 3:** Initial level and growth rates of linear predictors.

**Even growth patterns**				**Uneven growth patterns**		
	**A1**	**A2**	**A3**	**A1**	**A2**	**A3**
γ_00_	−1.38	−1.10	−0.85	−1.38	−1.10	−0.85
γ_01_	0.05	0.04	0.05	0	0.04	0.05

*A1, A2, and A3 represent Attribute 1, Attribute 2, and Attribute 3*.

##### Time variables

The current study planned to mimic the context of the interim assessments, which are administered several times within a school year (Great Schools Partnership, 2013). The common interval ranges from 6 to 8 weeks, such that individuals might receive the assessment at different times. Therefore, the current study set the time interval to 8 weeks and the unit of time to 1 week. The mean and standard deviation of time variables at each measurement occasion was fixed to μ_*time*_ = (0, 8, 16, 24, 32) and σ_*time*_ = 1, such that each individual had his/her own time variable at each measurement occasion to mimic the unbalanced data design.

As shown in Table 3, γ_00_ = −1.38 is the log-odds of the probability of 0.2, meaning at the first measurement occasion, the average probability of mastering Attribute 1 is 20%^2^. γ_01_ = 0.05 is growth rates of Attribute 1 in the log-odds scale, meaning that when time is increasing by one unit, the log-odds of probability of mastering Attribute 1 is increased by 0.05 in average, which is equal to the probability of mastery is increased by 0.008.

Table 4 presents the average base-rates of attributes across five measurement occasions, which was obtained by using the mean of the time variable and fixed effects shown in Table 3. Under the even growth pattern condition, the probabilities of mastery of three attributes were improved by 0.35, 0.38, and 0.39, respectively, across the time, and under the uneven pattern condition, the base-rate of Attribute 1 had a constant of 0.20, and the probabilities of mastery were improved by 0.38 and 0.39 for Attributes 2 and 3, respectively. This amount of improvement fell in the range of improvement of base-rates found in the previous studies (Li et al., 2016; Madison and Bradshaw, 2018).

**Table 4:** Base-rates of attributes over time.

	**T1**	**T2**	**T3**	**T4**	**T5**
**EVEN GROWTH PATTERN**					
A1	0.20	0.27	0.36	0.45	0.55
A2	0.25	0.32	0.40	0.49	0.58
A3	0.30	0.39	0.49	0.60	0.59
**UNEVEN GROWTH PATTERN**					
A1	0.20	0.20	0.20	0.20	0.20
A2	0.25	0.32	0.40	0.49	0.58
A3	0.30	0.39	0.49	0.60	0.59

*A1, A2, and A3 represent Attribute 1, Attribute 2, and Attribute 3; T1–T5 represent the first measurement occasion to the fifth measurement occasion*.

##### Item parameters

The intercepts of all items were fixed to −1.5 indicating the probability of having a correct answer was 0.18. The simple main effects of all items were fixed to 1.5, indicating the probability of having a correct answer was 0.50 given mastering this attribute. The interaction effects between two attributes were fixed to 0.50, indicating the probability of having a correct answer was 0.88, given mastering two attributes.

#### Data Generation Procedures

Data were generated in R, version 3.4.2 (R Core Team, 2017). Each condition was replicated 100 times.

Data generation procedures included two stages: first, the probability of mastery was generated for each attribute at five measurement occasions, then the mastery statuses of them was generated; lastly, the item response data was generated, which are proceeded as follows:

Generate the linear predictors of the probability of mastery for each attribute by using the intercept and slope parameters, time variables, and G matrix for each individual;
Convert this linear predictor into the probability of mastery;
A binary mastery status for each attribute is randomly drawn from the binomial distribution with the probability of mastering attributes.
Generate the probability of having a correct answer for each item using a prespecified Q-matrix, item parameters, and person profiles.
A binary item response is randomly sampled from the binomial distribution with the probability obtained from the last step.

### Analysis Plan and Outcome Variables

A Markov Chain Monte Carlo (MCMC) algorithm was adopted to estimate model parameters, which was implemented in the JAGS software (Plummer, 2003) by using the *R2jags* package (Su and Yajima, 2015) in the programming environment R (R Core Team, 2017). The JAGS syntax and more details of MCMC analyses can be found in the Supplementary Material.

The LCDM was applied to estimate the mastery statuses of attributes at each measurement occasion. For example, as described in the Q-matrix in Table 2, item 4 measured both Attribute 1 and Attribute 2. Thus, the probability of providing a correct answer to item 4 given the latent class *c* at Time *t* can be expressed as follows:

(8) $$\begin{array}{l} \pi_{4ct}=P\left({\text{x}}_{4\text{ct}}=1|\alpha_{\text{ct}}\right)=\\ \;\frac{\exp\left(\lambda_{4,0}+\lambda_{4,1,\left(1\right)}\left(\alpha_{1}\right)+\lambda_{4,1,\left(2\right)}\left(\alpha_{2}\right)+\lambda_{4,2,\left(1,2\right)}\left(\alpha_{1}\cdot \alpha_{2}\right)\right)}{1+\exp\left(\lambda_{4,0}+\lambda_{4,1,\left(1\right)}\left(\alpha_{1}\right)+\lambda_{4,1,\left(2\right)}\left(\alpha_{2}\right)+\lambda_{4,2,\left(1,2\right)}\left(\alpha_{1}\cdot \alpha_{2}\right)\right)} \end{array}$$

For items that only measure one attribute, only the intercept and the main effect of this item were included in the equation.

The generalized multivariate GCM was applied to measure the changes in mastery statuses of attributes over time. First, as suggested by MacCallum et al. (1997), Curran et al. (2012), and Hoffman (2015), a synthesized variable was created, which was a composite of multiple outcome variables ($\alpha_{rt}^{k}$ in the current study), then a series of dummy variables as exogenous predictors were adopted to control which specific outcomes were referenced within different parts of the model. Let *dv*_*rt*_ denote the synthesized variable, which contained individual *r*′*s* mastery statuses for three attributes across four measurement occasions. A total of three dummy variables, *A*1, *A*2, and *A*3, were included in the model to distinguish which specific element belonged to which specific outcome variables, where *A*1 was equal to 1 for Attribute 1 and *A*1 was equal to 0 for other attributes. Therefore, the probability of mastering attribute $\alpha_{rt}^{k}$ (*k* = 1, 2, 3) at time *t* could be described as follows:

(9) $$\begin{array}{c} logit\left(P\left(dv_{rt}=1\right)\right)=A1\left[\left(\gamma_{00}^{1}+u_{0r}^{1}\right)+(\gamma_{10}^{1}+u_{1r}^{1}\right)Time_{rt}]\\ +A2[(\gamma_{00}^{2}+u_{0r}^{2})+(\gamma_{10}^{2}+u_{1r}^{2})Time_{rt}]\\ +A3[(\gamma_{00}^{3}+u_{0r}^{3})+(\gamma_{10}^{3}+u_{1r}^{3})Time_{rt}] \end{array}$$

where the main effects of *A*1, *A*2, and *A*3 represent the initial levels for three attributes, and the interaction effects between dummy variables and time scores represent the growth rates for attributes.

Once data analysis was finished, the following outcome variables across all 100 replications were obtained for all conditions:

1. Gelman-Rubin diagnostic ($\hat{R}$) of parameters, including item parameters in the LCDM and both fixed effects and random effects parameters in the generalized growth curve model.
2. The distribution of estimated parameters, including the mean, standard deviation, and quantiles.

### Evaluation Criteria

Convergence rates, the classification accuracy of attributes at each measurement occasion, and the parameter recovery were evaluated in the current study to examine the performance of the proposed model under different conditions.

#### Convergence Rates

Convergence was assessed by using the Gelman-Rubin diagnostic ($\hat{R}$), also referred to as the “potential scale reduction factor” (Gelman and Rubin, 1992). Suppose there are *m* independent Markov chains, $\hat{R}$ is given by:

(10) $$\begin{array}{r} \sqrt{\hat{R}}=\sqrt{\frac{n-1}{n}+\frac{1}{n}\frac{B}{W}\;} \end{array}$$

where *B* is the variance between the means of the *m* chains, *W* is the average of the *m* within-chain variances, and *n* is the number of iterations of the chain after discarding the iterations as burn-in. If the algorithm converges, $\hat{R}$ is approaching 1, indicating a stationary distirbution has been achieved because the marginal posterior variance (weighted combo of between and within-chain variance) are equal to the within-chain variances. In the current study, $\hat{R}$ was calculated for all model parameters, and we adopted the criteria of $\hat{R}<1.2$ as the indicator of convergence as suggested by the previous study (e.g., Sinharay, 2003).

In one replication, if one or more parameters had the $\hat{R}$ larger than 1.2, this replication was regarded as non-converged. After a total of 100 replications, the convergence rates for this condition was calcualted and reported. Only the results from the converged replications were kept and used in the following analysis.

#### Classification Accuracy

The classification accuracy was evaluated by using (1) the bias of estimated probability of attribute mastery, (2) the correct classification rates for each mastery status, and (3) Cohen's kappa (Cohen, 1960).

The bias of the estimated probability of attribute mastery was the difference between the estimated and the true probability of attribute mastery. The correct classification rates for each mastery status included (1) the correct classification rates for individuals who truly mastered an attribute, and (2) the correct classification rates for individuals who truly did not master an attribute. Cohen's kappa measures the agreement between the true and the estimated mastery status.

The estimated class membership was obtained by applying 0.5 as the cutpoint, meaning that an individual with an estimated probability larger than 0.5 would be classified as mastery, vice versa.

#### Parameter Recovery

The bias and mean squared error (MSE) of estimated parameters, including item parameters from the measurement model, intercept and slope parameters, and variance and covariance parameters from the structural model were computed to assess the parameter recovery in each condition.

(11) $$Bias_{\theta }\;=\frac{\sum_{r\;=\;1}^{R}\sum_{i}^{N}\left({\hat{\theta }}_{ir}-\theta_{i}\right)}{RN}={\hat{\theta }}_{ir}-\theta_{i}$$

(12) $$\begin{array}{rcl} MSE_{\theta } & = & \frac{{\sum_{r\;=\;1}^{R}\sum_{i\;=\;1}^{N}\left({\hat{\theta }}_{ir}-\theta_{i}\right)}^{2}}{RN} \end{array}$$

where θ represents the estimated parameter, which is the mean of the sample distribution obtained from the Bayesian estimation. *R* is the number of replications; *N* is the number of elements in the set of θ.

A factorial analysis of variance was adopted to assess the impact of design factors on outcome variables. In all analyses, the α level was controlled at 0.05 level, and partial η^2^ was adopted as the measure of effect sizes. According to Cohen (1988) convention, partial η^2^ values of 0.01, 0.06, and 0.14 were regarded as small, medium, and large effects.

## Results

### Convergence Rates

As aforementioned, the Gelman-Rubin diagnostic ($\hat{R}$) of item parameters in the LCDM, fixed effects andrandom effects parameters in the generalized growth curve model were evaluated, and we adopted the criteria of $\hat{R}<1.2$ as the indicator of convergence as suggested by the previous study (e.g., Sinharay, 2003). When all the parameters, including the item parameters in the LCDM, fixed effects, and the random effects parameters in the generalized growth curve model were converged in one replication, this replication was regarded as converged. Results found that the average convergence rate is 0.95 under the conditions with three measurement occasions (*MO* = 3). And, the average convergence rate is 0.97 under the conditions with five measurement occasions (*MO* = 5). The details in convergence rates can be found in the Supplementary Material. Only the converged replications were used in the following analyses.

### Classification Accuracy

The classification accuracy was evaluated by using (1) bias of the estimated probability of attribute mastery, (2) correct classification rates for each mastery status, and (3) Cohen's kappa.

The average bias of probability of attribute mastery under the conditions when *MO* = 5 showed that the probability of attribute mastery was recovered well under most conditions. The average bias of the probability of attribute mastery was all close to 0 under most conditions. Similar patterns were found when *MO* = 3. For the sake of page limits, only the average bias from the condition *MO* = 5 in Table 5, the summary of *MO* = 3 could be found in the Supplementary Material.

**Table 5:** Bias of probability of attribute mastery (MO = 5).

			**T1**			**T2**			**T3**			**T4**			**T5**		
			**A1**	**A2**	**A3**	**A1**	**A2**	**A3**	**A1**	**A2**	**A3**	**A1**	**A2**	**A3**	**A1**	**A2**	**A3**
G1	gam1	N100	.	.	0.01	.	.	.	.	.	.	.	.	.	.	.	.
		N200	.	.	.	.	.	.	.	.	.	.	.	.	.	.	.
		N300	.	.	.	.	.	.	.	.	.	.	.	.	.	.	.
	gam2	N100	.	.	0.01	.	.	.	.	−0.01	.	.	.	.	.	.	.
		N200	.	.	.	.	.	.	.	.	.	.	.	.	.	.	.
		N300	.	.	.	.	.	.	.	.	.	.	.	.	.	.	.
G2	gam1	N100	.	0.01	.	.	.	.	.	.	.	.	.	.	.	.	.
		N200	.	.	.	.	.	.	.	.	.	.	.	.	.	.	.
		N300	.	0.01	.	.	.	.	.	.	.	.	.	.	.	.	.
	gam2	N100	0.01	.	0.01	.	−0.01	.	.	−0.01	.	.	−0.01	.	.	.	.
		N200	.	.	.	.	.	.	.	.	.	.	.	.	.	.	.
		N300	.	.	.	.	.	.	.	.	.	.	.	.	0.01	0.02	0.01

*T1–T5 represent the first to the fifth measurement occasion; A1, A2, and A3 represent Attribute 1, Attribute 2, and Attribute 3; N100, N200, and N300 represent the sample size of 100, 200, and 300, respectively; G1 and G2 represent equal correlation G and unequal correlation conditions of G matrix, respectively; gam1 and gam2 represent the same growth pattern across attributes and unequal growth patterns across attributes, respectively; · represents <0.001*.

Table 6 presents the average correct classification rates for individuals who truly mastered attributes, and Table 7 presents the correct classification rates for individuals who truly did not master attributes under different conditions when *MO* = 5. The average correct classification rates were very low for individuals who truly mastered the attributes at the first measurement occasion (*T* = 1), but the correct classification rates improved as the number of measurement occasions increased as shown in Table 6. For individuals who truly did not master the attributes, Table 7 shows that the correct classification rates were perfect at the first measurement occasion, and then decreased to about 0.9 at the following measurement occasions.

**Table 6:** Average correct classification rates for individuals who truly mastered attribute (MO = 5).

			**T1**			**T2**			**T3**			**T4**			**T5**		
			**A1**	**A2**	**A3**	**A1**	**A2**	**A3**	**A1**	**A2**	**A3**	**A1**	**A2**	**A3**	**A1**	**A2**	**A3**
G1	gam1	N100	0	0.04	0.06	0.69	0.73	0.76	0.86	0.88	0.88	0.90	0.91	0.92	0.92	0.94	0.94
		N200	0.03	0.02	0.03	0.70	0.73	0.77	0.86	0.87	0.89	0.91	0.91	0.92	0.93	0.93	0.94
		N300	0.02	0.02	0.03	0.70	0.73	0.77	0.87	0.87	0.88	0.91	0.91	0.91	0.93	0.93	0.94
	gam2	N100	0.04	0.04	0.07	0.60	0.71	0.76	0.80	0.86	0.89	0.87	0.90	0.92	0.90	0.92	0.93
		N200	0	0.02	0.03	0.62	0.73	0.77	0.82	0.86	0.89	0.88	0.91	0.92	0.90	0.93	0.94
		N300	0.01	0.02	0.02	0.64	0.73	0.77	0.82	0.88	0.88	0.88	0.91	0.92	0.90	0.93	0.94
G2	gam1	N100	0.05	0.08	0.07	0.69	0.73	0.75	0.87	0.87	0.88	0.91	0.91	0.91	0.93	0.94	0.93
		N200	0	0.03	0.04	0.70	0.73	0.76	0.86	0.87	0.88	0.90	0.91	0.92	0.92	0.93	0.94
		N300	0.02	0.02	0.03	0.69	0.73	0.76	0.86	0.86	0.88	0.90	0.90	0.91	0.91	0.92	0.93
	gam2	N100	0.05	0.04	0.07	0.64	0.70	0.75	0.82	0.86	0.87	0.88	0.91	0.92	0.90	0.92	0.94
		N200	0.02	0.02	0.04	0.63	0.73	0.77	0.83	0.87	0.89	0.87	0.91	0.91	0.90	0.93	0.94
		N300	0.02	0.02	0.02	0.64	0.74	0.76	0.83	0.87	0.88	0.88	0.91	0.91	0.90	0.93	0.93

*T1–T3 represent the first to the third measurement occasion; A1, A2, and A3 represent Attribute 1, Attribute 2, and Attribute 3; N100, N200, and N300 represent the sample size of 100, 200, and 300, respectively; G1 and G2 represent equal correlation G and unequal correlation conditions of G matrix, respectively; gam1 and gam2 represent the same growth pattern across attributes and unequal growth patterns across attributes, respectively*.

**Table 7:** Average correct classification rates for individuals who truly did not master attribute (MO = 5).

			**T1**			**T2**			**T3**			**T4**			**T5**		
			**A1**	**A2**	**A3**	**A1**	**A2**	**A3**	**A1**	**A2**	**A3**	**A1**	**A2**	**A3**	**A1**	**A2**	**A3**
G1	gam1	N100	1	1	1	0.87	0.83	0.79	0.88	0.87	0.85	0.92	0.90	0.89	0.94	0.94	0.93
		N200	1	1	1	0.86	0.83	0.79	0.87	0.87	0.86	0.92	0.91	0.90	0.94	0.94	0.93
		N300	1	1	1	0.86	0.82	0.79	0.87	0.87	0.86	0.91	0.91	0.90	0.94	0.94	0.93
	gam2	N100	1	1	0.99	0.91	0.84	0.79	0.91	0.87	0.86	0.94	0.91	0.90	0.96	0.94	0.93
		N200	1	1	1	0.91	0.83	0.79	0.91	0.87	0.85	0.94	0.91	0.89	0.95	0.93	0.93
		N300	1	1	1	0.90	0.82	0.78	0.90	0.87	0.85	0.93	0.91	0.90	0.96	0.94	0.93
G2	gam1	N100	1	1	0.99	0.86	0.82	0.80	0.86	0.86	0.85	0.91	0.90	0.90	0.94	0.93	0.93
		N200	1	1	1	0.85	0.82	0.79	0.87	0.86	0.85	0.91	0.91	0.89	0.94	0.94	0.93
		N300	1	1	1	0.85	0.82	0.79	0.86	0.85	0.85	0.91	0.90	0.89	0.93	0.92	0.92
	gam2	N100	1	1	0.99	0.90	0.85	0.80	0.90	0.86	0.85	0.94	0.91	0.90	0.96	0.94	0.94
		N200	1	1	1	0.90	0.82	0.79	0.90	0.87	0.86	0.94	0.92	0.90	0.95	0.94	0.93
		N300	1	1	1	0.89	0.82	0.79	0.90	0.87	0.85	0.93	0.91	0.89	0.94	0.92	0.91

*T1–T3 represent the first to the third measurement occasion; A1, A2, and A3 represent Attribute 1, Attribute 2, and Attribute 3; N100, N200, and N300 represent the sample size of 100, 200, and 300, respectively; G1 and G2 represent equal correlation G and unequal correlation conditions of G matrix, respectively; gam1 and gam2 represent the same growth pattern across attributes and unequal growth patterns across attributes, respectively*.

This pattern might be due to the cut point of 0.5 used in the current study. The true mastery status was randomly generated through a binomial distribution with the true probability of mastery, such that, there is still some probabilities of mastering attributes, even the probability is very low. However, the estimated probability of attribute mastery was very low on the first two measurement occasions; the majority of individuals' probabilities were lower than 0.5. After 0.5 was set as the cut point to classify individuals into mastery or non-mastery classes, most of the individuals were classified into the non-mastery class even they truly mastered the attributes by design. With the increasing of measurement occasions, the estimated probabilities for individuals who truly mastered the attributes were increasing to be larger than 0.5, thus the cut point of 0.5 can classify them correctly. Such that, the correct classification rate was very low on the first two measurement occasion, but it increases as the measurement occasions increase.

The similar patterns were found when *MO* = 3, which could be found in the Supplementary Material. In summary, even though the probability of attribute mastery were recovered well, the correct classification rates depended on the individuals' mastery status and the cut point that was adopted to classify individuals.

Cohen's kappa was calculated to evaluate the degree of agreement between the estimated and true mastery status. Table 8 presents the average kappa under different conditions when *MO* = 5. The calculation of kappa required that both true and estimated mastery status should have at least two levels; however, estimated mastery status only had one level under some conditions, especially at the first measurement occasion. Therefore, kappa was not applicable under some conditions. Results found that kappa values improved as time increased. This pattern might be due to the same reason as discussed above that the estimated probability of mastery was very low for all individuals at the first and second measurement occasions, such that after applying 0.5 as the cutpoint, the most of individuals who truly mastered the attributes were falsely classified to non-mastery. Therefore, kappa values were low at the beginning but improved as the number of measurement occasions increased. Similar patterns were found when *MO* = 3, which could be found in the Supplementary Material.

**Table 8:** Average kappa (MO = 5).

			**T1**			**T2**			**T3**			**T4**			**T5**		
			**A1**	**A2**	**A3**	**A1**	**A2**	**A3**	**A1**	**A2**	**A3**	**A1**	**A2**	**A3**	**A1**	**A2**	**A3**
G1	gam1	N100	.	.	.	0.57	0.55	0.55	0.74	0.75	0.72	0.82	0.81	0.81	0.87	0.88	0.86
		N200	.	.	.	0.56	0.56	0.56	0.73	0.74	0.74	0.82	0.82	0.82	0.87	0.87	0.87
		N300	.	.	.	0.56	0.55	0.56	0.73	0.73	0.74	0.83	0.82	0.82	0.87	0.87	0.87
	gam2	N100	.	.	.	0.54	0.55	0.55	0.72	0.72	0.75	0.82	0.81	0.82	0.86	0.86	0.86
		N200	.	.	.	0.56	0.56	0.56	0.73	0.73	0.73	0.82	0.82	0.81	0.86	0.86	0.87
		N300	.	.	.	0.55	0.55	0.55	0.73	0.74	0.74	0.82	0.82	0.82	0.86	0.87	0.87
G2	gam1	N100	.	.	.	0.56	0.55	0.55	0.72	0.72	0.73	0.82	0.81	0.81	0.87	0.86	0.87
		N200	.	.	.	0.56	0.55	0.55	0.73	0.73	0.74	0.82	0.82	0.81	0.87	0.87	0.86
		N300	.	.	.	0.54	0.55	0.55	0.72	0.71	0.73	0.80	0.80	0.80	0.85	0.84	0.85
	gam2	N100	.	.	.	0.56	0.55	0.55	0.72	0.72	0.73	0.82	0.82	0.82	0.86	0.86	0.87
		N200	.	.	.	0.54	0.55	0.55	0.73	0.74	0.75	0.81	0.83	0.82	0.85	0.87	0.87
		N300	.	.	.	0.54	0.55	0.54	0.73	0.74	0.73	0.81	0.81	0.80	.	.	.

*T1–T5 represent the first to the fifth measurement occasion; A1, A2, and A3 represent Attribute 1, Attribute 2, and Attribute 3; N100, N200, and N300 represent the sample size of 100, 200, and 300, respectively; G1 and G2 represent equal correlation G and unequal correlation conditions of G matrix, respectively; gam1 and gam2 represent the same growth pattern across attributes and unequal growth patterns across attributes, respectively; ‘.’ presents the kappa for this condition was not applicable*.

In summary, the agreement between true and estimated mastery status improved as the number of measurement occasions increased, and it was influenced by the cutpoint applied to classify individuals.

### Parameter Recovery

The bias and mean square error (MSE) of the estimated parameters were computed to assess the parameter recovery in each condition through the simulation. Then, ANOVA tests were conducted to assess the impact of the design factors on the bias and MSE values of the estimated parameters of the measurement model and the structural model, respectively.

#### Measurement Model Parameter Recovery

There were three sets of item parameters in the LCDM: the intercept (λ_0_), the main effect (λ_α_*k*__), and the interaction effect ($\lambda_{\alpha_{\text{k}}\alpha_{{\text{k}}^{\text{′}}}}$) parameters. Therefore, the average bias and MSE of all three sets of item parameters were assessed to evaluate the measurement model parameter recoveries.

As presented in Table 9, the proposed model achieved good parameter recoveries in intercept and main effect parameters, but the interaction parameters had relatively large bias and MSE values under most conditions. However, the recovery of the interaction effect parameters was improved as the sample size and the number of measurement occasions increased.

**Table 9:** Summary of measurement model parameter recoveries.

			**Three measurement occasions (MO = 3)**						**Five measurement occasions (MO = 5)**					
			**λ_**0**_**		**λ_**α**_***k***__**		$\lambda_{\alpha_{k}}{}_{\alpha_{k}{}^{\prime }}$		**λ_0_**		**λ_α_*k*__**		$\lambda_{\alpha_{k}}{}_{\alpha_{k}{}^{\prime }}$	
			**Bias**	**MSE**	**Bias**	**MSE**	**Bias**	**MSE**	**Bias**	**MSE**	**Bias**	**MSE**	**Bias**	**MSE**
G1	gam1	N100	0.03	0.05	−0.09	0.13	0.58	0.60	0.03	0.03	−0.07	0.07	0.31	0.24
		N200	0.02	0.03	−0.06	0.07	0.32	0.27	0.00	0.02	−0.03	0.04	0.15	0.11
		N300	0.02	0.02	−0.04	0.04	0.23	0.17	0.01	0.01	−0.03	0.03	0.11	0.07
	gam2	N100	0.04	0.05	−0.08	0.13	0.62	0.68	0.02	0.03	−0.07	0.07	0.33	0.27
		N200	0.03	0.03	−0.06	0.07	0.36	0.31	0.01	0.02	−0.03	0.04	0.16	0.11
		N300	0.01	0.02	−0.04	0.04	0.24	0.19	0.00	0.01	−0.02	0.03	0.09	0.07
G2	gam1	N100	0.04	0.05	−0.09	0.13	0.62	0.66	0.03	0.04	−0.07	0.07	0.31	0.25
		N200	0.02	0.03	−0.05	0.07	0.36	0.32	0.02	0.02	−0.04	0.04	0.17	0.11
		N300	0.01	0.02	−0.03	0.05	0.25	0.20	0.01	0.01	−0.02	0.03	0.10	0.07
	gam2	N100	0.03	0.05	−0.08	0.13	0.66	0.73	0.02	0.03	−0.06	0.07	0.34	0.28
		N200	0.03	0.03	−0.06	0.07	0.39	0.36	0.02	0.02	−0.04	0.04	0.18	0.13
		N300	0.01	0.02	−0.03	0.05	0.28	0.26	0.01	0.01	−0.02	0.03	0.11	0.08

*λ_0_, λ_α_k__, and $\lambda_{\alpha_{k}\alpha_{k^{\prime }}}$ represents the intercept, main effect, and interaction effect parameters of the LCDM; A1, A2, and A3 represent Attribute 1, Attribute 2, and Attribute 3; N100, N200, and N300 represent the sample size of 100, 200, and 300, respectively; G1 and G2 represent equal correlation G and unequal correlation conditions of G matrix, respectively; gam1 and gam2 represent the same growth pattern across attributes and unequal growth patterns across attributes, respectively*.

Since the bias and MSE values of item parameters were not consistent across conditions, ANOVA tests were conducted to examine the impact of design factors on them. When *MO* = 3, results found that the sample size had small to large effects on the recoveries on the intercept and main effects parameters ($\eta_{{\lambda_{0}}_{\text{Bias}}}^{2}=0.05,\;\eta_{{\lambda_{\alpha }}_{\text{Bias}}}^{2}=0.15;\;\eta_{{\lambda_{0}}_{MSE}}^{2}=0.67,\;\eta_{{\lambda_{\alpha }}_{\text{Bias}}}^{2}=0.74$). A large sample size was associated with good recoveries. The recoveries of interaction effect parameters were influenced by the sample size, the G matrix, and the growth pattern. The sample size had large effects on both the bias ($\eta_{{\lambda_{\alpha_{k}\alpha_{k^{{}^{\prime }}}}}_{bias}=}^{2}0.66$) and MSE ($\eta_{{\lambda_{\alpha_{k}\alpha_{k^{{}^{\prime }}}}}_{MSE}=}^{2}0.53$). Similarly, a large sample size resulted in better recoveries. Both the growth pattern and the G matrix design had small effects on interaction parameter recoveries (the growth pattern: $\eta_{{\lambda_{\alpha_{k}\alpha_{k^{{}^{\prime }}}}}_{bias}=}^{2}0.02,\;\eta_{{\lambda_{\alpha_{k}\alpha_{k^{{}^{\prime }}}}}_{MSE}=}^{2}0.02;$ the G matrix: $\eta_{{\lambda_{\alpha_{k}\alpha_{k^{{}^{\prime }}}}}_{bias}=}^{2}0.02,\;\eta_{{\lambda_{\alpha_{k}\alpha_{k^{{}^{\prime }}}}}_{MSE}=}^{2}0.02$); the growth and the equal correlations conditions resulted in better recoveries.

When *MO* = 5, the item parameter recoveries were mainly influenced by the sample size. The sample size had small to large effects on the recoveries of intercept and main effects ($\eta_{{\lambda_{0}}_{\text{Bias}}}^{2}=0.01,\;\eta_{{\lambda_{0}}_{\text{MSE}}}^{2}=0.33$; $\eta_{{\lambda_{\alpha }}_{\text{Bias}}}^{2}=0.05,\;\eta_{{\lambda_{\alpha }}_{\text{MSE}}}^{2}=0.37$), and large effects on the recoveries of interaction effects ($\eta_{{\lambda_{\alpha_{k}\alpha_{k^{{}^{\prime }}}}}_{bias}=}^{2}0.19,\;\eta_{{\lambda_{\alpha_{k}\alpha_{k^{{}^{\prime }}}}}_{MSE}=}^{2}0.17$). The parameter recoveries were improved as the sample size increased. In addition, the recoveries of intercept parameters were influenced by the growth pattern slightly. The non-growth condition had a slightly better intercept parameter recoveries, although the effect sizes were very small. For the sake of page limits, the details of ANOVA results could be found in the Supplementary Material.

In summary, the item parameter recoveries were mainly influenced by the sample size, especially for the interaction effect parameters. In general, the larger sample size resulted in the better item parameter recoveries.

#### Structural Model Parameter Recovery

Recoveries of both fixed effects and random effects in the growth model were evaluated in this study. The fixed effects included the intercept and slope parameters for each attribute ($\gamma_{00}^{{\text{A}}_{\text{k}}},\;\gamma_{01}^{{\text{A}}_{\text{k}}}$), and the random effects included the variance of intercept and slope parameters for each attribute ($\delta_{u_{0}^{A_{k}}},\;\delta_{u_{1}^{A_{k}}}$) as well as the covariance among intercept and slope parameters ($\delta_{u_{0}^{A_{k}},u_{0}^{A_{k^{{}^{\prime }}}}}\delta_{u_{1}^{A_{k}},u_{1}^{A_{k^{{}^{\prime }}}}}\delta_{u_{0}^{A_{k}},u_{1}^{A_{k^{{}^{\prime }}}}}$).

##### Recovery of the fixed effects

Table 10 presents the summary of average bias and MSE of fixed effects under all conditions when *MO* = 5, which reveals that the proposed model achieved good recoveries on the intercept parameters for Attributes 2 and 3, and slope parameters for all attributes, indicated by the small MSE values and the bias values being close to zero. However, the intercept parameter of Attribute 1 had relatively larger bias than other parameters. When *MO* = 3, similar patterns were found, which can be found in the Supplementary Material.

**Table 10:** Summary of fixed effects recoveries (MO = 5).

			$\gamma_{00}^{\text{A}1}$		$\gamma_{01}^{\text{A}1}$		$\gamma_{00}^{\text{A}2}$		$\gamma_{01}^{\text{A}2}$		$\gamma_{00}^{\text{A}3}$		$\gamma_{01}^{\text{A}3}$	
			**Bias**	**MSE**	**Bias**	**MSE**	**Bias**	**MSE**	**Bias**	**MSE**	**Bias**	**MSE**	**Bias**	**MSE**
G1	gam1	N100	−0.11	0.08	.	0.01	0.01	0.04	.	0.01	0.02	0.06	.	
		N200	−0.13	0.06	−0.01	.	0.02	0.05	.	.	0.05	0.05	.	.
		N300	−0.10	0.04	.	.	−0.01	0.03	0.01	.	0.03	0.04	−0.01	.
	gam2	N100	−0.12	0.08	0.01	0.01	0.01	0.06	.	0.01	0.01	0.05	.	0.01
		N200	−0.13	0.06	−0.02	0.01	0.02	0.05	0.01	.	0.05	0.06	0.01	0.01
		N300	−0.15	0.06	−0.01	.	.	0.04	0.01	.	0.10	0.04	−0.01	.
G2	gam1	N100	−0.14	0.09	−0.01	0.01	−0.01	0.07	.	0.01	0.07	0.08	0.01	0.01
		N200	−0.16	0.06	0.01	0.01	0.01	0.04	.	.	0.03	0.04	−0.01	0.01
		N300	−0.11	0.06	0.01	0.01	−0.02	0.04	0.01	.	0.01	0.03	−0.01	.
	gam2	N100	−0.12	0.07	−0.01	0.01	−0.03	0.07	−0.01	0.01	0.04	0.07	0.01	0.01
		N200	−0.13	0.07	0.01	.	−0.04	0.05	.	.	0.06	0.05	.	.
		N300	−0.11	0.05	.	.	−0.02	0.03	0.01	.	0.06	0.04	.	.

*$\gamma_{00}^{k}$ and $\gamma_{01}^{k}$ represents the intercept and slope parameters of attributes; A1, A2, and A3 represent Attribute 1, Attribute 2, and Attribute 3; N100, N200, and N300 represent the sample size of 100, 200, and 300, respectively; G1 and G2 represent equal correlation G and unequal correlation conditions of G matrix, respectively; gam1 and gam2 represent the same growth pattern across attributes and unequal growth patterns across attributes, respectively; represents <0.001*.

The bias and MSE of intercept parameters were not consistent across different conditions, so ANOVA tests were conducted to investigate if the design factors influenced the intercept parameter recoveries for both *MO* = 3 and *MO* = 5 conditions. As shown in Table 11, when *MO* = 3, the sample size had small effects on the MSE values of intercept parameters (${\eta^{2}}_{\gamma_{00}}^{A1}=0.03,\;{\eta^{2}}_{\gamma_{00}}^{A2}=0.04,\;{\eta^{2}}_{\gamma_{00}}^{A3}=0.03$). A large sample size was associated with small MSE values. However, the bias of fixed effects was not influenced by the design factors.

**Table 11:** ANOVA results of fixed effects parameter recoveries.

	**Three measurement occasions (MO = 3)**							**Five measurement occasions (MO = 5)**						
		**Bias**			**MSE**				**Bias**			**MSE**		
**Design factors**	**Df**	**F**	**η^2^**	**p**	**F**	**η^2^**	**P**	**df**	**F**	**η^2^**	**p**	**F**	**η^2^**	**p**
$\gamma_{00}^{A1}$														
G	1	0.1	.	0.75	0.37	.	0.54	1	0.05	.	0.82	1.05	.	0.31
SZ	2	0.06	.	0.94	14.93	0.03	.	1	2.04	.	0.15	1.11	.	0.29
G × SZ	2	0.42	.	0.66	0.83	.	0.43	1	1.19	.	0.28	0.11	.	0.74
Residuals	1128		0.5			0.5		770		0.5			0.5	
$\gamma_{00}^{A2}$														
G	1	0.89	.	0.35	2.78	.	0.1	1	2.57	.	0.11	0.37	.	0.55
SZ	2	0.72	.	0.49	22.14	0.04	.	1	1.02	.	0.31	8.58	0.01	.
G × SZ	2	0.05	.	0.95	0.23	.	0.79	1	0.22	.	0.64	0.08	.	0.78
Residuals	1128		0.5			0.5		770		0.5			0.5	
$\gamma_{00}^{A3}$														
G	1	2.31	.	0.13	0.28	.	0.6	1	1.26	.	0.26	0.42	.	0.52
SZ	2	0.03	.	0.97	15.78	0.03	.	1	0.2	.	0.65	6.65	0.01	0.01
G × SZ	2	1.07	.	0.34	1.46	.	0.23	1	0.38	.	0.54	1.4	.	0.24
Residuals	1128		0.5			0.5		770		0.5			0.5	

*G represents G matrix design; gamma represents the growth patterns; SZ represents the sample size; · represents <0.001*.

When *MO* = 5, ANOVA tests found that the sample size had small effects on the MSE values of intercept parameters for Attribute 2 and 3 (${\eta^{2}}_{\gamma_{00}}^{A2}=0.01,\;{\eta^{2}}_{\gamma_{00}}^{A3}=0.01$). Similarly, the bias of intercept parameters was not influenced by the design factors.

In summary, the intercept parameters of Attributes 2 and 3 and all the slope parameters were recovered well in the current study, but the intercept parameters of Attribute 1 had a relatively large bias. ANOVA tests found that the sample size had small effects on the MSE values of intercept parameters; a larger sample size resulted in smaller MSE values. However, no design factors were associated with the bias of intercept parameters.

##### Recovery of the random effects

Regarding the recovery of variance parameters, the average bias and MSE values of the variance of intercept and slope for all attributes were examined, the results reveal that the proposed model achieved good recoveries in both the intercept and slope variance parameters in both *MO* = 3 *and MO* = 5. The details of the summary of random variance recoveries could be found in the Supplementary Material.

Since bias of intercept variance parameters were not consistent across all conditions, ANOVA tests were conducted to examine the impact of design factors on them. As shown in Table 12, when *MO* = 3, results found that the sample size had medium effects on the bias of intercept variance parameters ($\eta_{\delta_{u_{0}^{A_{1}}}}^{2}=0.14;\;\eta_{\delta_{u_{0}^{A_{2}}}}^{2}=0.13;\;\eta_{\delta_{u_{0}^{A_{3}}}}^{2}=0.11$); the large sample size had large bias values.

**Table 12:** ANOVA results of random variance parameter recoveries.

	**Three measurement occasions (MO = 3)**							**Five measurement occasions (MO = 5)**						
		**Bias**			**MSE**				**Bias**			**MSE**		
**Design factors**	***Df***	***F***	***η^2^***	***p***	***F***	***η^2^***	***p***	***Df***	***F***	***η^2^***	***p***	***F***	***η^2^***	***p***
$\gamma_{00}^{A1}$														
G	1	0.10	.	0.75	1.53	.	0.22	1	2.21	.	0.14	.	.	0.97
SZ	2	89.68	0.14	.	1.27	0.02	.	1	14.49	0.02	.	2.35	.	0.13
G × SZ	2	2.58	.	0.08	1.12	.	0.33	1	0.01	.	0.94	0.19	.	0.66
Residuals	1128		0.50			0.50		770		0.50			0.50	
$\gamma_{00}^{A2}$														
G	1	0.08	.	0.78	2.79	.	0.10	1	.	.	0.97	0.54	.	0.46
SZ	2	84.06	0.13	.	11.79	0.02	.	1	13.35	0.02	.	4.22	0.01	0.04
G × SZ	2	1.78	.	0.17	2.19	.	0.11	1	0.01	.	0.92	1.48	.	0.22
Residuals	1128		0.50			0.50		770		0.50			0.50	
$\gamma_{00}^{A3}$														
G	1	0.14	.	0.71	3.54	.	0.06	1	0.64	.	0.43	.	.	1.
SZ	2	72.67	0.11	.	7.26	0.01	.	1	14.08	0.02	.	6.48	0.01	0.01
G × SZ	2	3.01	0.01	0.05	2.37	.	0.09	1	0.08	.	0.77	0.59	.	0.44
Residuals	1128		0.50			0.50		770		0.50			0.50	

*G represents G matrix design; gamma represents the growth patterns; SZ represents the sample size; · represents <0.001*.

When *MO* = 5, similar patterns were found. The variance of intercept and slope parameters were recovered well. Since the recoveries of the variance of intercept parameters were varied by conditions, ANOVA tests were conducted to investigate the impact of design factors on them. As showed in Table 12, the sample size had small effects ($\eta_{\delta_{u_{0}^{A_{1}}}}^{2}=0.02;\;\eta_{\delta_{u_{0}^{A_{2}}}}^{2}=0.02;\;\eta_{\delta_{u_{0}^{A_{3}}}}^{2}=0.02$); the larger sample size had larger bias values.

In summary, the proposed model achieved good recoveries on the variance of intercept and slope parameters. Moreover, a large sample size was associated with large bias values of the variance of intercept parameters.

Regarding the recovery of covariance parameters, on average, the proposed model achieved good recoveries on the covariance among intercept and slope parameters for both *MO* = 5 and *MO* = 3. However, the covariance between intercepts had a lightly larger bias than other sets of parameters. Details of the summary of covariance parameter recoveries could be found in the Supplementary Material.

When *MO* = 3, As shown in Table 13, ANOVA tests found that the sample size had medium effects (η^2^ = 0.13) on the bias of covariance between intercept parameters; a large sample size was associated with a large bias.

**Table 13:** ANOVA results of random covariance parameter recoveries.

	**Three measurement occasions (MO = 3)**							**Five measurement occasions (MO = 5)**						
		**Bias**			**MSE**				**Bias**			**MSE**		
**Design factors**	**Df**	**F**	**η^2^**	**p**	**F**	**η^2^**	**p**	**df**	**F**	**η^2^**	**p**	**F**	**η^2^**	**p**
$\delta_{u_{0}^{A_{k}},u_{0}^{A_{k^{{}^{\prime }}}}}$														
G	1	0.16	.	0.69	3.2	.	0.07	1	0.82	.	0.37	0.1	.	0.75
SZ	2	81.22	0.13	.	6.96	0.01	.	1	5.32	0.01	0.02	5.81	0.01	0.02
G × SZ	2	2.87	0.01	0.06	2.04	.	0.13	1	0.25	.	0.62	0.7	.	0.4
Residuals	1128		0.5				0.5		770		0.5		0.5	

*G represents G matrix design; gamma represents the growth patterns; SZ represents the sample size; · represents <0.001*.

Similar patterns were found when *MO* = 5, ANOVA tests showed the sample size had medium effects on the bias values of covariance between intercept parameters; a larger sample size was associated with a larger bias value.

On average, the proposed model achieved good recoveries on the covariance among intercept and slope parameters. The bias of covariance among intercept parameters was influenced by the sample size; the larger sample size resulted in larger bias values.

## Discussion

### Performance of the Multivariate Longitudinal DCM

#### Model Convergence

Overall, the proposed model achieved satisfactory convergence rates; however, the proposed achieved a slightly higher convergence rates when *MO* = 5 than *MO* = 3, which was reasonable since more measurement occasions would provide more information to help the estimation and the model be converged. Also, as shown in the Supplementary Material, the conditions with five measurement occasions had more chains and a longer chain length for each chain than the conditions with three measurement occasions, which might have led to an improvement in the convergence rates. Therefore, the number of chains and the chain length might be not sufficient for the conditions with three measurement occasions.

#### Classification Accuracy

The bias of the estimated probability of attribute mastery, the correct classification rates for each mastery status, and Cohen's kappa was used to evaluate the classification accuracy of the proposed model.

The probability of attribute mastery was recovered well in the current study consistently across all measurement occasions, which indicated that the proposed model could provide accurate estimates of probabilities of attribute mastery.

Regarding correct classification rates, results found different patterns for individuals who truly mastered the attributes and individuals who truly did not master the attributes. For the individuals who truly mastered the attributes, the correct classification rates improved significantly as the number of measurement occasions increased. However, for individuals who truly did not master the attributes, the correct classification rates decreased slightly as the number of measurement occasions increased. This pattern might be due to that we adopted the cut point of 0.5 to classify the individuals. Since the estimated probabilities of attribute mastery for most of the individuals were lower than 0.5 on the first two measurement occasions, individuals would be classified into the non-mastery status, even some of them truly mastered the attributes by design. As a result, the correct classification rates were low for individuals who truly mastered the attributes on the first two measurement occasions. As the number of measurement occasions increased, the estimated probability of mastery increased, such that correct classification rates increased. Due to the same reason, Cohen's kappa increased as the number of measurement occasions increased. Therefore, the cutpoint had influenced the correct classification rates and kappa values of the current model.

#### Parameter Recoveries

The bias and mean square error (MSE) of the estimated parameters were computed to assess the parameter recovery in each condition through the simulation.

##### Measurement model parameter recoveries

Regarding the item parameter recoveries, conditions with three and five measurement occasions illustrated similar patterns. The proposed model achieved good parameter recoveries in intercept and main effect parameters, but poor interaction effect parameter recoveries. However, the recoveries of the interaction effect parameters were improved as the sample size and the number of measurement occasions increased. In addition, results from the ANOVA tests found the sample size had large impact on the interaction effects recoveries. Nonetheless, this result was expected. Previous research showed that the intercept and main effect parameters were easier to recover than the two-way interaction effect parameters. The recoveries of the interaction effect parameters were problematic when the sample size was <1,000 (e.g., Choi et al., 2010; Kunina-Habenicht et al., 2012). Therefore, these results suggested that a large sample size was necessary to achieve good item parameter recoveries in the LCDM framework, especially for the interaction effect parameters. The maximum sample size (*n* = 300) in the current study was not sufficient for obtaining accurate interaction effect parameters, especially for the conditions with three measurement occasions.

##### Structural model parameter recoveries

Both the recoveries of fixed effects and random effects in the generalized growth curve model were evaluated.

Regarding the recoveries of the fixed effects, overall, the proposed model achieved good intercept recoveries for Attributes 2 and 3, and slope recoveries for all attributes, but relatively poor recoveries for Attribute 1 intercept. Attribute 1 had relatively small intercept value by design ($\gamma_{00}^{A1}=-1.38$), therefore, the small intercept value might have led to enlarge the bias. To avoid the influence of the small value of the intercept parameter, the time variable could be centered at the medial measurement occasions (*T* = 2 when *MO* = 3, or *T* = 3 when *MO* = 5), such that there would be sufficient information to estimate the intercept parameters.

Regarding the recoveries of the random effects, on average, the proposed model achieved good recoveries on the random effects, including the variance of intercept and slope parameters of each attribute as well as the covariance among intercept and slope parameters within and crossed attributes. To improve the model convergence, the current study adopted the true variance-covariance matrix in the population as the prior of the estimated variance-covariance matrix, which might have led to good recoveries of the random effects.

### Conclusion and Recommendations

The current study developed a multivariate longitudinal DCM that could measure growth in attributes over time, and it evaluated this proposed model using a simulation study. The results revealed the following: (1) In general, the proposed model provided good convergence rates under different conditions. (2) Regarding the classification accuracy, the proposed model achieved good recoveries on the probabilities of attribute mastery. For individuals who truly mastered the attributes, the correct classification rates increased as the measurement occasions increased; however, for individuals who truly did not master the attributes, the correct classification rates decreased slightly as the numbers of measurement occasions increased. Cohen's kappa increased as the number of measurement occasions increased. (3) Both the intercept and main effect parameters in the LCDM were recovered well. The interaction effect parameters had a relatively large bias under the condition with a small sample size and fewer measurement occasions; however, the recoveries were improved as the sample size and the number of measurement occasions increased. (4) Overall, the proposed model achieved acceptable recoveries on both the fixed and random effects in the generalized growth curve model.

In summary, a large sample size is recommended for applying the proposed model to the real data. When the sample size is small, the scale with a simple structure of the Q matrix is recommended, because the interaction effects in the LCDM might not be estimated accurately with the small sample size. Also, applied researchers are suggested to center the time variable at the medial measurement occasion to improve the recovery of the intercept parameter in the generalized growth curve model. Additionally, when doing the MCMC analysis, multiple chains with the longer chain length are recommended to achieve satisfied model convergence rates.

Therefore, when practitioners try to measure students' growth in the DCM framework using the proposed model, they should use a larger sample size, an assessment with less complex Q-matrix design, and multiple chains with longer chain length to maximize the convergence rates and the accuracy of parameter estimates.

### Contributions and Limitations

In the current study, a multivariate longitudinal DCM was developed to analyze longitudinal data under the DCM framework. It represents an improvement in the current longitudinal DCMs given its ability to incorporate both balanced and unbalanced data and to measure the growth of a single attribute directly without assuming that attributes grow in the same pattern. The current study had several limitations. First, the true variance-covariance matrix was used as the prior for the random effects parameters in the generalized growth curve model in the current study; however, the true variance-covariance matrix is unknown when fitting the model to the real data. Therefore, future studies could adopt a non-informative variance-covariance matrix as the prior, then evaluate if the proposed model could achieve satisfying recoveries on the random effects as well. Second, local item dependency was not incorporated in the current study. However, in real longitudinal data, repeated measures always have some degree of local item dependence (e.g., Cai, 2010). Therefore, future research could simulate local item dependence with the common items to mimic real data. Third, only three or five measurement occasions were included in the current model. The small number of measurement occasions might have limited the reliability and accuracy of the estimation of the growth curve model (e.g., Finch, 2017). In the future, more measurement occasions could be included to examine the performance of the proposed model comprehensively. Fourth, the definition of the time variable in longitudinal studies is very crucial. In the current study, we follow a conventional way to use the length of time between adjacent measurement occasions as the time variable. However, in reality, students likely have spent different lengths of time learning different attributes. So, in the future, we suggest using the number of hours spent on learning an attribute as the time variable if the data is available. In addition, we applied the cut-score to the average of the post burn-in probability of master to obtain a binary master status of one iteration on each condition, meaning that we cannot obtain a posterior distribution of the mastery status. So, we suggest future researchers applying the cut-score within MCMC analysis to obtain a posterior distribution of mastery status, which should provide a more accurate estimated mastery status. Last but not least, due to the limited data resources, we did not find a real dataset to evaluate the proposed model. We plan to add a real data application if some longitudinal diagnose assessment data is available in the future.

## Data Availability Statement

All datasets generated for this study are included in the article/Supplementary Material.

## Author Contributions

QP drafted the manuscript, conducted, and interpreted the statistical analyses. LQ reviewed the manuscript and provided expertise on data analyses. NK supervised and reviewed the paper. All authors contributed to the article and approved the submitted version.

## Conflict of Interest

The authors declare that the research was conducted in the absence of any commercial or financial relationships that could be construed as a potential conflict of interest.

## Footnotes

^1^In the current study, the balanced data refers to equal time intervals and unbalanced data refers to unequal time intervals.

^2^This equation describes the relationships between the log odds of probability and fixed effects. $\log\left(\frac{probability}{1-probability}\right)=\log\left(\frac{0.2}{1-0.2}\right)=-1.38$.
