# Supplementary material for: Growth Modeling in a Diagnostic Classification Model (DCM) Framework–A Multivariate Longitudinal Diagnostic Classification Model
Source: Front Psychol. 2020 Aug 7;11:1714. doi: 10.3389/fpsyg.2020.01714 (PMC7438873; doi:10.3389/fpsyg.2020.01714)
Supplement: Supplementary file 1 [file Table_1.DOCX]

Supplementary Material

# Supplementary Data

The R code for generating one example dataset in the current study is listed below:

## load packages ##

library('MASS')

library('truncnorm')

library('rjags')

library('R2jags')

library('lavaan')

library('MBESS')

library('mnormt')

## set seed ##

set.seed(12345)

## generation starts ##

n.chains = 4

n.iter = 25000

n.cluster= n.chains # n.chains = n.cluster

# G1gamm1 T3 SZ 100

s <- Sys.time()

# desgin factor variation #

T <- 3;N = 100

g = 1; gam = 1

# g = 1; gam = 2

# g = 2; gam = 1

# g = 2; gam = 2

# factor 1 #######

# gamma matrix #

prob_linearpred <- function(prob){

linearpred <- as.data.frame(log(prob/(1-prob)))

return(linearpred)

}

# generate gamma matrix #####

gen_gamma <- function(intercept_slope_matrix){

intercept <- intercept_slope_matrix$V1

slope <- (intercept_slope_matrix$V2 - intercept_slope_matrix$V1)/32

intercept_slope <- as.data.frame(cbind(intercept,slope))

return(intercept_slope)

}

prob_list <- vector('list',2)

# even growth

prob_list[[1]] <- matrix(c(0.2,0.25,0.3,0.55,0.58,0.69),3,2)

# uneven growth

prob_list[[2]] <- matrix(c(0.2,0.25,0.3,0.2,0.58,0.69),3,2)

t1_t4_linearpred_list <- lapply(prob_list,prob_linearpred)

# intercept and slopes

int_slope_list <- lapply(t1_t4_linearpred_list, gen_gamma)

##########################################################

# factor 2 #####

# G matrix

# same correlation among attributes

G<- vector('list',2)

lower <- '

1,

0.2, 1,

0.9, 0.1, 1,

0.1, 0.25, 0.2, 1,

0.9, 0.1 , 0.9, 0.1, 1,

0.1, 0.25, 0.1, 0.25, 0.2, 1'

cor <- getCov(lower,names=c('A1u0','A1u1','A2u0','A2u1','A3u0','A3u1'))

var <- c(0.15,0.05,0.15,0.05,0.15,0.05)

sd <- sqrt(var)

cov <- cor2cov(cor,sd)

cov

G[[1]] <- cov

# diff correlation among attributes

lower <- '

1,

0.2, 1,

0.15, 0.01, 1,

0.01, 0.01, 0.20, 1,

0.10, 0.01, 0.20, 0.10, 1,

0.01, 0.01, 0.10, 0.25, 0.20, 1'

cor <- getCov(lower,names=c('A1u0','A1u1','A2u0','A2u1','A3u0','A3u1'))

var <- c(0.15,0.05,0.15,0.05,0.15,0.05)

sd <- sqrt(var)

cov <- cor2cov(cor,sd)

cov

G[[2]] <- cov

#######################################

# fixed conditions

# Q <- read.csv(file = 'Qmatrix.csv',header = T)[,-1]

Q <- c(

1, 0, 0,

0, 1, 0,

0, 0, 1,

1, 1, 0,

1, 0, 1,

0, 1, 1,

1, 0, 0,

0, 1, 0,

0, 0, 1,

1, 1, 0,

1, 0, 1,

0, 1, 1,

1, 0, 0,

0, 1, 0,

0, 0, 1,

1, 1, 0,

1, 0, 1,

0, 1, 1,

1, 0, 0,

0, 1, 0,

0, 0, 1,

1, 1, 0,

1, 0, 1,

0, 1, 1,

1, 0, 0,

0, 1, 0,

0, 0, 1,

1, 1, 0,

1, 0, 1,

0, 1, 1)

Q <- matrix(Q,3,30)

Q <- as.data.frame(t(Q))

I <- dim(Q)[1]

K <- dim(Q)[2]

# item parameters

lam0= rep(-1.5,30)

lam1= rep(1.5,30)

lam2= rep(1.5,30)

lam3= rep(1.5,30)

interaction= 0.5

T1 <- round(rnorm(n=N,mean = 0,sd=1),0)

T2 <- round(rnorm(n=N,mean = 8,sd=1),0)

T3 <- round(rnorm(n=N,mean = 16,sd=1),0)

PersonTime <- as.data.frame(cbind(T1,T2, T3))

colnames(PersonTime) <- c('T1','T2','T3')

####################################################

## 1. generate mastery status----

# gamma matrix

gamma <- as.data.frame(t(int_slope_list[[gam]]))

colnames(gamma)<-c('Att1','Att2','Att3')

rownames(gamma) <- c('gamma0','gamma1')

# G matrix

cov <- G[[g]]

u <- mvrnorm(n=N,mu=c(0,0,0,0,0,0),Sigma = cov,empirical = F)

cov <- cov(u) # true cov in this round

## Person Attribute Mastery Generation ##

### Attribute 1 #####

PersonAtt1 <- as.data.frame(matrix(0,N,9))

colnames(PersonAtt1) <- c('A1T1','A1T2','A1T3',

'A1T1_prob','A1T2_prob','A1T3_prob',

'A1T1_linearpred','A1T2_linearpred','A1T3_linearpred')

for (person in 1:N){

# time 1

# person=1

u0 <- u[person,1]

u1 <- u[person,2]

Att1_t1_linear_predictor <- gamma$Att1[1]+u0 + (gamma$Att1[2]+u1)*PersonTime$T1[person]

Att1_t1_probability <- exp(Att1_t1_linear_predictor)/(1+exp(Att1_t1_linear_predictor))

Att1_t1 <- rbinom(n = 1, size = 1, prob = Att1_t1_probability)

PersonAtt1$A1T1_prob[person] = Att1_t1_probability

PersonAtt1$A1T1_linearpred[person] = Att1_t1_linear_predictor

PersonAtt1$A1T1[person] = Att1_t1

# time 2

Att1_t2_linear_predictor <- gamma$Att1[1]+u0 + (gamma$Att1[2]+u1)*PersonTime$T2[person]

Att1_t2_probability <- exp(Att1_t2_linear_predictor)/(1+exp(Att1_t2_linear_predictor))

Att1_t2 <- rbinom(n = 1, size = 1, prob = Att1_t2_probability)

PersonAtt1$A1T2_prob[person] = Att1_t2_probability

PersonAtt1$A1T2_linearpred[person] = Att1_t2_linear_predictor

PersonAtt1$A1T2[person] = Att1_t2

# time 3

Att1_t3_linear_predictor <- gamma$Att1[1]+u0 + (gamma$Att1[2]+u1)*PersonTime$T3[person]

Att1_t3_probability <- exp(Att1_t3_linear_predictor)/(1+exp(Att1_t3_linear_predictor))

Att1_t3 <- rbinom(n = 1, size = 1, prob = Att1_t3_probability

PersonAtt1$A1T3_prob[person] = Att1_t3_probability

PersonAtt1$A1T3_linearpred[person] = Att1_t3_linear_predictor

PersonAtt1$A1T3[person] = Att1_t3

}

### Attribute 2 ####

PersonAtt2 <- as.data.frame(matrix(0,N,9))

colnames(PersonAtt2) <- c('A2T1','A2T2','A2T3',

'A2T1_prob','A2T2_prob','A2T3_prob',

'A2T1_linearpred','A2T2_linearpred','A2T3_linearpred')

for (person in 1:N){

# time 1

# person=1

u0 <- u[person,3]

u1 <- u[person,4]

Att2_t1_linear_predictor <- gamma$Att2[1]+u0 + (gamma$Att2[2]+u1)*PersonTime$T1[person]

Att2_t1_probability <- exp(Att2_t1_linear_predictor)/(1+exp(Att2_t1_linear_predictor))

Att2_t1 <- rbinom(n = 1, size = 1, prob = Att2_t1_probability)

PersonAtt2$A2T1_prob[person] = Att2_t1_probability

PersonAtt2$A2T1_linearpred[person] = Att2_t1_linear_predictor

PersonAtt2$A2T1[person] = Att2_t1

# time 2

Att2_t2_linear_predictor <- gamma$Att2[1]+u0 + (gamma$Att2[2]+u1)*PersonTime$T2[person]

Att2_t2_probability <- exp(Att2_t2_linear_predictor)/(1+exp(Att2_t2_linear_predictor))

Att2_t2 <- rbinom(n = 1, size = 1, prob = Att2_t2_probability)

PersonAtt2$A2T2_prob[person] = Att2_t2_probability

PersonAtt2$A2T2_linearpred[person] = Att2_t2_linear_predictor

PersonAtt2$A2T2[person] = Att2_t2

# time 3

Att2_t3_linear_predictor <- gamma$Att2[1]+u0 + (gamma$Att2[2]+u1)*PersonTime$T3[person]

Att2_t3_probability <- exp(Att2_t3_linear_predictor)/(1+exp(Att2_t3_linear_predictor))

Att2_t3 <- rbinom(n = 1, size = 1, prob = Att2_t3_probability)

PersonAtt2$A2T3_prob[person] = Att2_t3_probability

PersonAtt2$A2T3_linearpred[person] = Att2_t3_linear_predictor

PersonAtt2$A2T3[person] = Att2_t3

}

### Attribute 3 ####

PersonAtt3 <- as.data.frame(matrix(0,N,9))

colnames(PersonAtt3) <- c('A3T1','A3T2','A3T3',

'A3T1_prob','A3T2_prob','A3T3_prob',

'A3T1_linearpred','A3T2_linearpred','A3T3_linearpred')

for (person in 1:N){

# time 1

# person=1

u0 <- u[person,5]

u1 <- u[person,6]

Att3_t1_linear_predictor <- gamma$Att3[1]+u0 + (gamma$Att3[2]+u1)*PersonTime$T1[person]

Att3_t1_probability <- exp(Att3_t1_linear_predictor)/(1+exp(Att3_t1_linear_predictor))

Att3_t1 <- rbinom(n = 1, size = 1, prob = Att3_t1_probability)

PersonAtt3$A3T1_prob[person] = Att3_t1_probability

PersonAtt3$A3T1_linearpred[person] = Att3_t1_linear_predictor

PersonAtt3$A3T1[person] = Att3_t1

# time 2

Att3_t2_linear_predictor <-gamma$Att3[1]+u0 + (gamma$Att3[2]+u1)*PersonTime$T2[person]

Att3_t2_probability <- exp(Att3_t2_linear_predictor)/(1+exp(Att3_t2_linear_predictor))

Att3_t2 <- rbinom(n = 1, size = 1, prob = Att3_t2_probability)

PersonAtt3$A3T2_prob[person] = Att3_t2_probability

PersonAtt3$A3T2_linearpred[person] = Att3_t2_linear_predictor

PersonAtt3$A3T2[person] = Att3_t2

# time 3

Att3_t3_linear_predictor <- gamma$Att3[1]+u0 + (gamma$Att3[2]+u1)*PersonTime$T3[person]

Att3_t3_probability <- exp(Att3_t3_linear_predictor)/(1+exp(Att3_t3_linear_predictor))

Att3_t3 <- rbinom(n = 1, size = 1, prob = Att3_t3_probability)

PersonAtt3$A3T3_prob[person] = Att3_t3_probability

PersonAtt3$A3T3_linearpred[person] = Att3_t3_linear_predictor

PersonAtt3$A3T3[person] = Att3_t3

}

### generate Item response data ####

true.profile_T1 <- as.data.frame(cbind(PersonAtt1$A1T1,PersonAtt2$A2T1,PersonAtt3$A3T1))

true.profile_T2 <- as.data.frame(cbind(PersonAtt1$A1T2,PersonAtt2$A2T2,PersonAtt3$A3T2))

true.profile_T3 <- as.data.frame(cbind(PersonAtt1$A1T3,PersonAtt2$A2T3,PersonAtt3$A3T3))

# time 1 data----

nitems <- I

PersonData = NULL

DatamatBinary = matrix(data = NA, nrow = N, ncol = nitems)

for(person in 1:N){

# generate person data

PersonProfile <- true.profile_T1[person,]

PersonData = rbind(PersonData, PersonProfile)

for(item in 1:nitems){

linear_predictor <- lam0[item] + Q[item,1]*lam1[item]*PersonProfile[1] +

Q[item,2]*lam2[item]*PersonProfile[2] + Q[item,3]*lam3[item]*PersonProfile[3]+

Q[item,1]*Q[item,2]*interaction*PersonProfile[1]*PersonProfile[2] +

Q[item,1]*Q[item,3]*interaction*PersonProfile[1]*PersonProfile[3] +

Q[item,2]*Q[item,3]*interaction*PersonProfile[2]*PersonProfile[3]

#convert linear predictor probability using inverse logit link function

item_probability = exp(linear_predictor)/(1+exp(linear_predictor))

#draw random value

DatamatBinary[person, item] = rbinom(n = 1, size = 1, prob = as.numeric(item_probability))

}

# print(person)

}

colnames(DatamatBinary) <- c(paste0('I',1:30))

DatamatBinary_T1 <- DatamatBinary

#############

# time 2 data-------

PersonData = NULL

DatamatBinary = matrix(data = NA, nrow = N, ncol = nitems)

for(person in 1:N){

# generate person data

# person=1

PersonProfile <- true.profile_T2[person,]

PersonData = rbind(PersonData, PersonProfile)

for(item in 1:nitems){

# item=4

linear_predictor <- lam0[item] + Q[item,1]*lam1[item]*PersonProfile[1] +

Q[item,2]*lam2[item]*PersonProfile[2] + Q[item,3]*lam3[item]*PersonProfile[3]+

Q[item,1]*Q[item,2]*interaction*PersonProfile[1]*PersonProfile[2] +

Q[item,1]*Q[item,3]*interaction*PersonProfile[1]*PersonProfile[3] +

Q[item,2]*Q[item,3]*interaction*PersonProfile[2]*PersonProfile[3]

#convert linear predictor probability using inverse logit link function

item_probability = exp(linear_predictor)/(1+exp(linear_predictor))

#draw random value

DatamatBinary[person, item] = rbinom(n = 1, size = 1, prob = as.numeric(item_probability))

}

# print(person)

}

colnames(DatamatBinary) <- c(paste0('I',1:30))

DatamatBinary_T2 <- DatamatBinary

########

#time 3 data-----

PersonData = NULL

DatamatBinary = matrix(data = NA, nrow = N, ncol = nitems)

for(person in 1:N){

# generate person data

# person=1

PersonProfile <- true.profile_T3[person,]

PersonData = rbind(PersonData, PersonProfile)

for(item in 1:nitems){

# item=4

linear_predictor <- lam0[item] + Q[item,1]*lam1[item]*PersonProfile[1] +

Q[item,2]*lam2[item]*PersonProfile[2] + Q[item,3]*lam3[item]*PersonProfile[3]+

Q[item,1]*Q[item,2]*interaction*PersonProfile[1]*PersonProfile[2] +

Q[item,1]*Q[item,3]*interaction*PersonProfile[1]*PersonProfile[3] +

Q[item,2]*Q[item,3]*interaction*PersonProfile[2]*PersonProfile[3]

#convert linear predictor probability using inverse logit link function

item_probability = exp(linear_predictor)/(1+exp(linear_predictor))

#draw random value

DatamatBinary[person, item] = rbinom(n = 1, size = 1, prob = as.numeric(item_probability))

}

# print(person)

}

colnames(DatamatBinary) <- c(paste0('I',1:30))

DatamatBinary_T3 <- DatamatBinary

# Supplementary Tables

Table 2.1 presents the average convergence rates for all conditions, indicating the proposed model achieved good convergence rates across conditions.

Table 2.1. Average Convergence Rates

|  |  | Three Measurement Occasions  ($MO=3$) | | |  | Five Measurement Occasions  ($MO=5$) | | |
| --- | --- | --- | --- | --- | --- | --- | --- | --- |
|  |  | N100 | N200 | N300 |  | N100 | N200 | N300 |
| G1 | gam1 | .91 | .97 | .96 |  | .98 | 1 | .90 |
|  | gam2 | .88 | .95 | .91 |  | .98 | .99 | .95 |
| G2 | gam1 | .93 | .97 | .99 |  | .98 | .98 | .92 |
|  | gam2 | .91 | .97 | .99 |  | 1 | 1 | 1 |

Note. N100, N200, and N300 represent the sample size of 100, 200, and 300, respectively; G1 and G2 represent equal correlation G and unequal correlation conditions of G matrix, respectively; gam1 and gam2 represent the same growth pattern across attributes and unequal growth patterns across attributes respectively.

Table 2.2 to Table 2.6 show the classification accuracy of the proposed model under the condition with three measurement occasions, including the bias of probability of attribute mastery, the average correct classification rates for individuals who truly mastered attributes and who truly did not master attributes, and the average kappa.

Table 2.2. The Bias of Probability of Attribute Mastery (MO=3)

|  |  | T1 | | | T2 | | | T3 | | |
| --- | --- | --- | --- | --- | --- | --- | --- | --- | --- | --- |
|  |  | A1 | A2 | A3 | A1 | A2 | A3 | A1 | A2 | A3 |
| gam1 | N100 | . | . | . | -.01 | . | -.01 | . | . | . |
|  | N200 | . | . | . | -.01 | -.01 | . | . | . | .01 |
|  | N300 | . | . | . | . | -.01 | . | . | . | -.01 |
| gam2 | N100 | . | . | . | -.01 | -.01 | -.01 | -.01 | . | -.01 |
|  | N200 | -.01 | . | . | -.01 | . | . | -.01 | . | . |
|  | N300 | . | . | . | -.01 | -.01 | . | . | -.01 | . |
| gam1 | N100 | . | . | -.01 | -.01 | -.01 | -.01 | -.01 | . | . |
|  | N200 | . | . | . | -.01 | . | . | . | . | . |
|  | N300 | . | . | . | . | . | . | . | . | . |
| gam2 | N100 | -.01 | . | -.01 | -.01 | . | -.01 | -.01 | . | . |
|  | N200 | . | . | -.01 | -.01 | . | -.01 | . | . | . |
|  | N300 | . | . | .01 | -.01 | -.01 | . | . | . | . |

Note. T1 to T3 represent the first to the third measurement occasion; A1, A2, and A3 represent Attribute 1, Attribute 2, and Attribute 3; N100, N200, and N300 represent the sample size of 100, 200, and 300, respectively; G1 and G2 represent equal correlation G and unequal correlation conditions of G matrix, respectively; gam1 and gam2 represent the same growth pattern across attributes and unequal growth patterns across attributes, respectively; . represents < .001.rage

Table 2.3. The MSE of Probability of Attribute Mastery (MO=3)

|  |  | T1 | | | T2 | | | T3 | | |
| --- | --- | --- | --- | --- | --- | --- | --- | --- | --- | --- |
|  |  | A1 | A2 | A3 | A1 | A2 | A3 | A1 | A2 | A3 |
| gam1 | N100 | .01 | .01 | .01 | .04 | .04 | .04 | .07 | .07 | .07 |
|  | N200 | .01 | .01 | .01 | .04 | .04 | .04 | .07 | .07 | .07 |
|  | N300 | . | . | . | -.01 | -.01 | -.01 | . | . | -.01 |
| gam2 | N100 | .01 | .01 | .01 | .04 | .04 | .04 | .07 | .07 | .07 |
|  | N200 | .01 | .01 | .01 | .03 | .04 | .04 | .07 | .07 | .07 |
|  | N300 | . | . | . | -.01 | -.01 | . | -.01 | -.01 | . |
| gam1 | N100 | .01 | .01 | .01 | .04 | .04 | .05 | .07 | .07 | .07 |
|  | N200 | .01 | .01 | .01 | .04 | .04 | .04 | .07 | .07 | .07 |
|  | N300 | . | . | . | . | . | . | . | .01 | . |
| gam2 | N100 | .01 | .01 | .01 | .04 | .04 | .05 | .07 | .07 | .07 |
|  | N200 | .01 | .01 | .01 | .04 | .04 | .04 | .07 | .07 | .07 |
|  | N300 | -.01 | . | .01 | -.01 | -.01 | . | . | . | . |

Note. T1 to T3 represent the first to the third measurement occasion; A1, A2, and A3 represent Attribute 1, Attribute 2, and Attribute 3; N100, N200, and N300 represent the sample size of 100, 200, and 300, respectively; G1 and G2 represent equal correlation G and unequal correlation conditions of G matrix, respectively; gam1 and gam2 represent the same growth pattern across attributes and unequal growth patterns across attributes, respectively; . represents < .001.rage Attribute-wise Classification

Table 2.4. Average Correct Classification Rates for Individuals Who Truly Mastered Attribute (MO=3)

|  |  |  | T1 | | | T2 | | | T3 | | |
| --- | --- | --- | --- | --- | --- | --- | --- | --- | --- | --- | --- |
|  |  |  | A1 | A2 | A3 | A1 | A2 | A3 | A1 | A2 | A3 |
| G1 | gam1 | N100 | .04 | .06 | .08 | .61 | .65 | .70 | .86 | .88 | .89 |
|  |  | N200 | .03 | .03 | .04 | .63 | .69 | .75 | .87 | .89 | .91 |
|  |  | N300 | .02 | .02 | .03 | .64 | .69 | .73 | .88 | .88 | .89 |
|  | gam2 | N100 | .06 | .05 | .08 | .53 | .66 | .71 | .80 | .87 | .88 |
|  |  | N200 | .03 | .03 | .04 | .52 | .69 | .73 | .79 | .88 | .90 |
|  |  | N300 | .04 | .02 | .02 | .55 | .68 | .74 | .81 | .88 | .91 |
| G2 | gam1 | N100 | 0 | .06 | .11 | .62 | .64 | .73 | .85 | .87 | .89 |
|  |  | N200 | .02 | .02 | .04 | .64 | .67 | .73 | .87 | .88 | .90 |
|  |  | N300 | .02 | .02 | .03 | .66 | .70 | .74 | .88 | .89 | .91 |
|  | gam2 | N100 | .04 | .06 | .06 | .50 | .66 | .70 | .78 | .86 | .87 |
|  |  | N200 | .02 | .02 | .03 | .54 | .69 | .71 | .81 | .88 | .90 |
|  |  | N300 | .02 | .02 | .02 | .55 | .69 | .74 | .82 | .89 | .90 |

Note. T1 to T3 represent the first to the third measurement occasion; A1, A2, and A3 represent Attribute 1, Attribute 2, and Attribute 3; N100, N200, and N300 represent the sample size of 100, 200, and 300, respectively; G1 and G2 represent equal correlation G and unequal correlation conditions of G matrix, respectively; gam1 and gam2 represent the same growth pattern across attributes and unequal growth patterns across attributes, respectively.

Table 2.5. Average Correct Classification Rates for Individuals Who Truly did not Mastered Attribute (MO=3)

|  |  |  | T1 | | | T2 | | | T3 | | |
| --- | --- | --- | --- | --- | --- | --- | --- | --- | --- | --- | --- |
|  |  |  | A1 | A2 | A3 | A1 | A2 | A3 | A1 | A2 | A3 |
| G1 | gam1 | N100 | 1 | 1 | .99 | .94 | .91 | .85 | .93 | .92 | .91 |
|  |  | N200 | 1 | 1 | 1 | .94 | .90 | .84 | .94 | .93 | .91 |
|  |  | N300 | 1 | 1 | 1 | .94 | .90 | .85 | .94 | .93 | .92 |
|  | gam2 | N100 | 1 | 1 | 1 | .96 | .90 | .84 | .96 | .92 | .90 |
|  |  | N200 | 1 | 1 | 1 | .97 | .90 | .84 | .97 | .92 | .92 |
|  |  | N300 | 1 | 1 | 1 | .97 | .90 | .84 | .97 | .94 | .91 |
| G2 | gam1 | N100 | 1 | 1 | .99 | .95 | .90 | .86 | .94 | .93 | .90 |
|  |  | N200 | 1 | 1 | 1 | .95 | .91 | .84 | .94 | .93 | .91 |
|  |  | N300 | 1 | 1 | 1 | .95 | .90 | .84 | .93 | .93 | .92 |
|  | gam2 | N100 | 1 | 1 | 1 | .97 | .90 | .86 | .96 | .93 | .91 |
|  |  | N200 | 1 | 1 | 1 | .97 | .90 | .85 | .96 | .92 | .91 |
|  |  | N300 | 1 | 1 | 1 | .97 | .91 | .84 | .96 | .93 | .92 |

Note. T1 to T3 represent the first to the third measurement occasion; A1, A2, and A3 represent Attribute 1, Attribute 2, and Attribute 3; N100, N200, and N300 represent the sample size of 100, 200, and 300, respectively; G1 and G2 represent equal correlation G and unequal correlation conditions of G matrix, respectively; gam1 and gam2 represent the same growth pattern across attributes and unequal growth patterns across attributes, respectively.

Table 2.6. Average Kappa (MO=3)

|  |  | T1 | | | T2 | | | T3 | | |
| --- | --- | --- | --- | --- | --- | --- | --- | --- | --- | --- |
|  |  | A1 | A2 | A3 | A1 | A2 | A3 | A1 | A2 | A3 |
| gam1 | N100 | . | . | . | .58 | .58 | .55 | .80 | .80 | .80 |
|  | N200 | . | . | . | .60 | .60 | .59 | .81 | .83 | .82 |
|  | N300 | . | . | . | .61 | .60 | .58 | .83 | .82 | .82 |
| gam2 | N100 | . | . | . | .55 | .57 | .56 | .79 | .79 | .79 |
|  | N200 | . | . | . | .56 | .60 | .57 | .79 | .81 | .82 |
|  | N300 | . | . | . | .58 | .60 | .58 | .80 | .82 | .82 |
| gam1 | N100 | . | . | . | . | .57 | .59 | .80 | .80 | .80 |
|  | N200 | . | . | . | .63 | .60 | .58 | .82 | .81 | .81 |
|  | N300 | . | . | . | .64 | .62 | .58 | .82 | .82 | .83 |
| gam2 | N100 | . | . | . | .53 | .58 | .56 | .76 | .80 | .79 |
|  | N200 | . | . | . | .58 | .61 | .57 | .79 | .80 | .80 |
|  | N300 | . | . | . | .58 | .62 | .58 | .81 | .82 | .82 |

Note. T1 to T3 represent the first to the third measurement occasion; A1, A2, and A3 represent Attribute 1, Attribute 2, and Attribute 3; N100, N200, and N300 represent the sample size of 100, 200, and 300, respectively; G1 and G2 represent equal correlation G and unequal correlation conditions of G matrix, respectively; gam1 and gam2 represent the same growth pattern across attributes and unequal growth patterns across attributes, respectively; . presents the kappa for this condition was not applicable.

Table 2.7 presents the MSE of probability of attribute mastery across five measurement occacsions.

Table 2.7. The MSE of Probability of Attribute Mastery (MO=5)

|  |  |  | T1 | | | T2 | | | T3 | | | T4 | | | T5 | | |
| --- | --- | --- | --- | --- | --- | --- | --- | --- | --- | --- | --- | --- | --- | --- | --- | --- | --- |
|  |  |  | A1 | A2 | A3 | A1 | A2 | A3 | A1 | A2 | A3 | A1 | A2 | A3 | A1 | A2 | A3 |
| G1 | gam1 | N100 | .01 | .01 | .01 | .02 | .02 | .02 | .03 | .03 | .03 | .03 | .03 | .03 | .04 | .03 | .04 |
|  |  | N200 | .01 | .01 | .01 | .02 | .02 | .02 | .03 | .03 | .03 | .03 | .03 | .03 | .03 | .04 | .03 |
|  |  | N300 | . | .01 | .01 | .02 | .02 | .02 | .03 | .03 | .03 | .03 | .03 | .03 | .03 | .03 | .03 |
|  | gam2 | N100 | .01 | .01 | .01 | .02 | .02 | .02 | .03 | .03 | .03 | .03 | .03 | .03 | .04 | .04 | .04 |
|  |  | N200 | . | .01 | .01 | .02 | .02 | .02 | .03 | .03 | .03 | .03 | .03 | .03 | .04 | .03 | .04 |
|  |  | N300 | . | .01 | .01 | .02 | .02 | .02 | .03 | .03 | .03 | .03 | .03 | .03 | .04 | .04 | .03 |
| G2 | gam1 | N100 | .01 | .01 | .01 | .02 | .02 | .02 | .03 | .03 | .03 | .03 | .03 | .03 | .04 | .04 | .04 |
|  |  | N200 | .01 | .01 | .01 | .02 | .02 | .02 | .03 | .03 | .03 | .03 | .03 | .03 | .04 | .04 | .04 |
|  |  | N300 | . | .01 | .01 | .02 | .03 | .03 | .03 | .03 | .03 | .04 | .04 | .04 | .04 | .04 | .04 |
|  | gam2 | N100 | .01 | .01 | .01 | .02 | .02 | .02 | .03 | .03 | .03 | .03 | .03 | .03 | .04 | .04 | .04 |
|  |  | N200 | .01 | .01 | .01 | .02 | .02 | .02 | .03 | .03 | .03 | .03 | .03 | .03 | .04 | .04 | .03 |
|  |  | N300 | . | .01 | .01 | .02 | .02 | .02 | .03 | .03 | .03 | .03 | .03 | .04 | .05 | .05 | .05 |

Note. T1 to T5 represent the first to the fifth measurement occasion; A1, A2, and A3 represent Attribute 1, Attribute 2, and Attribute 3; N100, N200, and N300 represent the sample size of 100, 200, and 300, respectively; G1 and G2 represent equal correlation G and unequal correlation conditions of G matrix, respectively; gam1 and gam2 represent the same growth pattern across attributes and unequal growth patterns across attributes, respectively; . represents < .001.rage

Table 2.8 shows the recoveries of fixed effects under the condition of three measurement occasions.

Table 2.8. Summary of Fixed Effects Recoveries (MO=3)

|  |  |  | $\gamma_{00}^{A1}$ | | $\gamma_{01}^{A1}$ | | $\gamma_{00}^{A2}$ | | $\gamma_{01}^{A2}$ | | $\gamma_{00}^{A3}$ | | $\gamma_{01}^{A3}$ | |
| --- | --- | --- | --- | --- | --- | --- | --- | --- | --- | --- | --- | --- | --- | --- |
|  |  |  | Bias | MSE | Bias | MSE | Bias | MSE | Bias | MSE | Bias | MSE | Bias | MSE |
| G1 | gam1 | N100 | -.10 | .11 | . | .01 | -.02 | .08 | . | .01 | .07 | .10 | -.02 | .01 |
|  |  | N200 | -.11 | .08 | -.01 | .01 | .01 | .07 | . | .01 | .03 | .08 | . | .01 |
|  |  | N300 | -.11 | .05 | . | . | .02 | .06 | . | .01 | .04 | .06 | -.01 | . |
|  | gam2 | N100 | -.11 | .11 | . | .01 | .09 | .11 | .02 | .01 | .06 | .10 | . | .01 |
|  |  | N200 | -.13 | .07 | .01 | .01 | -.01 | .06 | .02 | .01 | .06 | .07 | . | .01 |
|  |  | N300 | -.10 | .06 | -.01 | . | .02 | .04 | . | . | .05 | .05 | . | . |
| G2 | gam1 | N100 | -.12 | .09 | -.01 | .01 | . | .09 | . | .01 | .02 | .08 | . | .01 |
|  |  | N200 | -.09 | .08 | .01 | .01 | -.01 | .05 | . | .01 | .05 | .07 | .01 | .01 |
|  |  | N300 | -.11 | .06 | .01 | . | .02 | .05 | .01 | .01 | .02 | .06 | . | . |
|  | gam2 | N100 | -.11 | .10 | -.01 | .01 | .03 | .09 | .02 | .01 | . | .14 | . | .01 |
|  |  | N200 | -.11 | .06 | . | .01 | . | .06 | . | .01 | .02 | .05 | .02 | .01 |
|  |  | N300 | -.11 | .07 | .01 | . | -.01 | .04 | . | . | .05 | .05 | . | .01 |

Note. $\gamma_{00}^{k}$ and $\gamma_{01}^{k}$ represents the intercept and slope parameters of attributes; A1, A2, and A3 represent Attribute 1, Attribute 2, and Attribute 3; N100, N200, and N300 represent the sample size of 100, 200, and 300, respectively; . represents < .001.

Table 2.9 shows the summary of ANOVA results of measurement model parameter recoveries.

Table 2.9. ANOVA Results of Measurement Model Parameter Recoveries

| Three Measurement Occasions (MO=3) | | | | | | | |  | Five Measurement Occasions (MO=5) | | | | | | |
| --- | --- | --- | --- | --- | --- | --- | --- | --- | --- | --- | --- | --- | --- | --- | --- |
|  |  | Bias | | | MSE | | |  |  | Bias | | | MSE | | |
| $\lambda_{0}$ |  |  |  |  |  |  |  |  |  |  |  |  |  |  |  |
| Design factors | *df* | *F* | 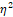 | *p* | *F* | 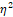 | *p* |  | *df* | *F* | 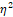 | *p* | *F* | 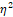 | *p* |
| G | 1 | . | . | 0.99 | 2.73 | . | 0.1 |  | 1 | 6.25 | 0.01 | 0.01 | 5.87 | 0.01 | 0.02 |
| gamma | 1 | 0.45 | . | 0.5 | 1.7 | . | 0.19 |  | 1 | 0.15 | . | 0.7 | 7.66 | 0.01 | 0.01 |
| SZ | 2 | 29.46 | 0.05 | . | 1156.49 | 0.67 | . |  | 1 | 4.96 | 0.01 | 0.03 | 384.5 | 0.33 | . |
| G$\times$gamma | 1 | 0.07 | . | 0.79 | 0.1 | . | 0.75 |  | 1 | 0.01 | . | 0.93 | 2.17 | . | 0.14 |
| G$\times$SZ | 2 | 0.21 | . | 0.81 | 0.19 | . | 0.83 |  | 1 | 5.82 | 0.01 | 0.02 | 0.21 | . | 0.65 |
| gamma$\times$SZ | 2 | 0.62 | . | 0.54 | 2.16 | . | 0.12 |  | 1 | 1.78 | . | 0.18 | 1.12 | . | 0.29 |
| G$\times$gamma$\times$SZ | 2 | 1.95 | . | 0.14 | 0.27 | . | 0.77 |  | 1 | 4.24 | 0.01 | 0.04 | 4.7 | 0.01 | 0.03 |
| Residuals | 1122 |  | 0.5 |  |  | 0.5 |  |  | 766 |  | 0.5 |  |  | 0.5 |  |
| $\lambda_{\alpha_{k}}$ |  |  |  |  |  |  |  |  |  |  |  |  |  |  |  |
| Design factors | *df* | *F* | 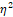 | *p* | *F* | 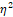 | *p* |  | df | F | 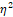 | p | F | 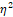 | p |
| G | 1 | 1.13 | . | 0.29 | 0.01 | . | 0.92 |  | 1 | 1.33 | . | 0.25 | 0.33 | . | 0.57 |
| gamma | 1 | 1.94 | . | 0.16 | 1.14 | . | 0.29 |  | 1 | 2.2 | . | 0.14 | 1.68 | . | 0.2 |
| SZ | 2 | 96.38 | 0.15 | . | 1641.27 | 0.75 | . |  | 1 | 38.92 | 0.05 | . | 454.47 | 0.37 | . |
| G$\times$gamma | 1 | 0.02 | . | 0.9 | 0.11 | . | 0.74 |  | 1 | 0.09 | . | 0.77 | 1.37 | . | 0.24 |
| G$\times$SZ | 2 | 0.1 | . | 0.9 | 0.16 | . | 0.86 |  | 1 | 7.44 | 0.01 | 0.01 | 0.17 | . | 0.68 |
| gamma$\times$SZ | 2 | 1.89 | . | 0.15 | 0.82 | . | 0.44 |  | 1 | 0.37 | . | 0.55 | 0.1 | . | 0.76 |
| G$\times$gamma$\times$SZ | 2 | 0.35 | . | 0.71 | 1.54 | . | 0.21 |  | 1 | 2.9 | . | 0.09 | 2.2 | . | 0.14 |
| Residuals | 1122 |  | 0.5 |  |  | 0.5 |  |  | 766 |  | 0.5 |  |  | 0.5 |  |
| $\lambda_{\alpha_{k}\alpha_{k'}}$ |  |  |  |  |  |  |  |  |  |  |  |  |  |  |  |
| Design factors | *df* | *F* | 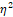 | *p* | *F* | 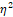 | *p* |  | df | F | 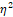 | p | F | 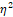 | p |
| G | 1 | 27.97 | 0.02 | . | 24.8 | 0.02 | . |  | 1 | 6.75 | 0.01 | 0.01 | 5.93 | 0.01 | 0.02 |
| gamma | 1 | 23.81 | 0.02 | . | 21.05 | 0.02 | . |  | 1 | 0.6 | . | 0.44 | 4.7 | 0.01 | 0.03 |
| SZ | 2 | 1078.32 | 0.66 | . | 644.66 | 0.53 | . |  | 1 | 176.94 | 0.19 | . | 158.16 | 0.17 | . |
| G$\times$gamma | 1 | 0.57 | . | 0.45 | 0.19 | . | 0.66 |  | 1 | 4.86 | 0.01 | 0.03 | 2.27 | . | 0.13 |
| G$\times$SZ | 2 | 0.7 | . | 0.49 | 0.06 | . | 0.94 |  | 1 | 0.85 | . | 0.36 | 0.82 | . | 0.37 |
| gamma$\times$SZ | 2 | 0.72 | . | 0.49 | 1.14 | . | 0.32 |  | 1 | 0.81 | . | 0.37 | 2.27 | . | 0.13 |
| G$\times$gamma$\times$SZ | 2 | 0.47 | . | 0.63 | 0.48 | . | 0.62 |  | 1 | 2 | . | 0.16 | 0.02 | . | 0.89 |
| Residuals | 1122 |  | 0.5 |  |  | 0.5 |  |  | 766 |  | 0.5 |  |  | 0.5 |  |

*Note.* G represents G matrix design; gamma represents the growth patterns; SZ represents the sample size; . represents < .001

Table 2.10 to Table 2.12 present the summary of recoveries of random effects.

Table 2.10. Summary of Random Variance Recoveries (MO=3)

|  |  |  | $\delta_{u_{0}^{A_{1}}}$ | | $\delta_{u_{0}^{A_{2}}}$ | | $\delta_{u_{0}^{A_{3}}}$ | | $\delta_{u_{1}^{A_{1}}}$ | | $\delta_{u_{1}^{A_{2}}}$ | | $\delta_{u_{1}^{A_{3}}}$ | |
| --- | --- | --- | --- | --- | --- | --- | --- | --- | --- | --- | --- | --- | --- | --- |
|  |  |  | Bias | MSE | Bias | MSE | Bias | MSE | Bias | MSE | Bias | MSE | Bias | MSE |
| G1 | gam1 | N100 | .02 | .01 | .03 | .01 | .02 | .01 | . | . | . | . | .01 | . |
|  |  | N200 | -.01 | . | -.01 | . | . | . | . | . | . | . | . | . |
|  |  | N300 | -.03 | . | -.03 | . | -.03 | . | . | . | . | . | . | . |
|  | gam2 | N100 | .02 | . | .02 | . | .01 | . | .01 | . | .01 | . | .01 | . |
|  |  | N200 | -.02 | . | -.01 | . | -.01 | . | . | . | . | . | . | . |
|  |  | N300 | -.03 | . | -.03 | . | -.03 | . | . | . | . | . | . | . |
| G2 | gam1 | N100 | .04 | .01 | .04 | .01 | .04 | .01 | .01 | . | .01 | . | .01 | . |
|  |  | N200 | -.01 | . | -.01 | . | -.01 | .01 | . | . | . | . | . | . |
|  |  | N300 | -.04 | . | -.03 | . | -.03 | . | . | . | . | . | . | . |
|  | gam2 | N100 | .03 | .01 | .03 | .01 | .03 | .01 | .01 | . | .01 | . | . | . |
|  |  | N200 | -.02 | . | -.02 | . | -.02 | . | .01 | . | . | . | . | . |
|  |  | N300 | -.03 | . | -.03 | . | -.03 | . | . | . | . | . | . | . |

Note: A1, A2, and A3 represent Attribute 1, Attribute 2, and Attribute 3; N100, N200, and N300 represent the sample size of 100, 200, and 300, respectively; G1 and G2 represent equal correlation G and unequal correlation conditions of G matrix, respectively; gam1 and gam2 represent the same growth pattern across attributes and unequal growth patterns across attributes, respectively; . represents < .001.

Table 2.11. Summary of Random Variance Recoveries (MO=5)

|  |  |  | $\delta_{u_{0}^{A_{1}}}$ | | $\delta_{u_{0}^{A_{2}}}$ | | $\delta_{u_{0}^{A_{3}}}$ | | $\delta_{u_{1}^{A_{1}}}$ | | $\delta_{u_{1}^{A_{2}}}$ | | $\delta_{u_{1}^{A_{3}}}$ | |
| --- | --- | --- | --- | --- | --- | --- | --- | --- | --- | --- | --- | --- | --- | --- |
|  |  |  | Bias | MSE | Bias | MSE | Bias | MSE | Bias | MSE | Bias | MSE | Bias | MSE |
| G1 | gam1 | N100 | . | . | . | . | -.01 | . | .01 | . | .01 | . | . | . |
|  |  | N200 | -.03 | . | -.03 | . | -.03 | . | . | . | . | . | . | . |
|  |  | N300 | -.04 | . | -.04 | . | -.04 | . | . | . | . | . | . | . |
|  | gam2 | N100 | .01 | .01 | .01 | . | . | . | . | . | . | . | . | . |
|  |  | N200 | -.03 | . | -.03 | . | -.04 | . | . | . | . | . | . | . |
|  |  | N300 | -.04 | . | -.04 | . | -.04 | . | . | . | . | . | . | . |
| G2 | gam1 | N100 | . | . | . | . | . | . | .01 | . | . | . | .01 | . |
|  |  | N200 | -.04 | . | -.03 | . | -.04 | . | . | . | . | . | . | . |
|  |  | N300 | -.05 | . | -.04 | . | -.04 | . | . | . | . | . | . | . |
|  | gam2 | N100 | . | . | -.01 | . | -.01 | . | .01 | . | . | . | .01 | . |
|  |  | N200 | -.03 | . | -.03 | . | -.03 | . | . | . | . | . | . | . |
|  |  | N300 | -.05 | . | -.05 | . | -.05 | . | . | . | . | . | . | . |

*Note:* A1, A2, and A3 represent Attribute 1, Attribute 2, and Attribute 3; N100, N200, and N300 represent the sample size of 100, 200, and 300, respectively; G1 and G2 represent equal correlation G and unequal correlation conditions of G matrix, respectively; gam1 and gam2 represent the same growth pattern across attributes and unequal growth patterns across attributes, respectively; . represents < .001.

Table 2.12. Summary of Covariance Parameter Recoveries

|  |  |  | Three Measurement Occasions (MO=3) | | | | | |  | Five Measurement Occasions (MO=5) | | | | | |
| --- | --- | --- | --- | --- | --- | --- | --- | --- | --- | --- | --- | --- | --- | --- | --- |
|  |  |  | $\delta_{u_{0}^{A_{k}},u_{0}^{A_{k^{'}}}}$ | | $\delta_{u_{1}^{A_{k}},u_{1}^{A_{k^{'}}}}$ | | $\delta_{u_{0}^{A_{k}},u_{1}^{A_{k^{'}}}}$ | |  | $\delta_{u_{0}^{A_{k}},u_{0}^{A_{k^{'}}}}$ | | $\delta_{u_{1}^{A_{k}},u_{1}^{A_{k^{'}}}}$ | | $\delta_{u_{0}^{A_{k}},u_{1}^{A_{k^{'}}}}$ | |
|  |  |  | Bias | MSE | Bias | MSE | Bias | MSE |  | Bias | MSE | Bias | MSE | Bias | MSE |
| G1 | gam1 | N100 | .02 | .01 | . | . | . | . |  | -.01 | . | . | . | . | . |
|  |  | N200 | -.01 | . | . | . | . | . |  | -.04 | . | . | . | . | . |
|  |  | N300 | -.03 | . | . | . | . | . |  | -.05 | . | . | . | . | . |
|  | gam2 | N100 | .01 | . | . | . | . | . |  | . | . | . | . | . | . |
|  |  | N200 | -.02 | . | . | . | . | . |  | -.04 | . | . | . | . | . |
|  |  | N300 | -.03 | . | . | . | . | . |  | -.05 | . | . | . | . | . |
| G2 | gam1 | N100 | .03 | .01 | . | . | . | . |  | -.01 | . | . | . | . | . |
|  |  | N200 | -.02 | . | . | . | . | . |  | -.04 | . | . | . | . | . |
|  |  | N300 | -.04 | . | . | . | . | . |  | -.05 | . | . | . | .01 | . |
|  | gam2 | N100 | .02 | .01 | . | . | . | . |  | -.02 | . | . | . | . | . |
|  |  | N200 | -.03 | . | . | . | . | . |  | -.04 | . | . | . | . | . |
|  |  | N300 | -.03 | . | . | . | . | . |  | -.05 | . | . |  | . |  |

Note: A1, A2, and A3 represent Attribute 1, Attribute 2, and Attribute 3; N100, N200, and N300 represent the sample size of 100, 200, and 300, respectively; G1 and G2 represent equal correlation G and unequal correlation conditions of G matrix, respectively; gam1 and gam2 represent the same growth pattern across attributes and unequal growth patterns across attributes, respectively; . represents < .001.

# Supplementary JAGS code

Jags code for the multivariate longitudinal DCM is listed below:

model{

# the LCDM

for(t in 1:T){ # time points

for(n in 1:N){ # people

for(i in 1:I){ # item

logit(p[n, i, t]) <- lamda0[i] + lamda1[i] * alpha[n,1,t] * Q[i,1] + lamda2[i] * alpha[n,2,t] * Q[i,2] + lamda3[i] * alpha[n,3,t] * Q[i,3] + lamda12[i] * alpha[n,1,t] * Q[i,1] * alpha[n,2,t] * Q[i,2] + lamda13[i] * alpha[n,1,t] * Q[i,1] * alpha[n,3,t] * Q[i,3] + lamda23[i] * alpha[n,2,t] * Q[i,2] * alpha[n,3,t] * Q[i,3]

Y[n, i, t] ~ dbern(p[n, i, t])}

}}

## growth model

for(t in 1:T){

for(n in 1:N){

logit(prob[n,1,t]) <- gamma[1,1]+ u[n,1] + (gamma[1,2] + u[n,2])*PersonTime[n,t]

logit(prob[n,2,t]) <- gamma[2,1]+ u[n,3] + (gamma[2,2] + u[n,4])*PersonTime[n,t]

logit(prob[n,3,t]) <- gamma[3,1]+ u[n,5] + (gamma[3,2] + u[n,6])*PersonTime[n,t]

alpha[n,1,t] ~ dbern(prob[n,1,t])

alpha[n,2,t] ~ dbern(prob[n,2,t])

alpha[n,3,t] ~ dbern(prob[n,3,t])

}}

# prior for the growth model

# for random effects

for( n in 1:N){

u[n,1:6] ~ dmnorm(mean.u[1:6],latprec.u[1:6,1:6])

}

for(g in 1:6){

mean.u[g] ~ dnorm(0,.0001)

}

latprec.u[1:6,1:6] ~ dwish(cov.u,6)

latcov.u[1:6,1:6] <- inverse(latprec.u[1:6,1:6])

# for fixed effects

gamma[1,1] ~ dnorm(mean.int[1],.001)

gamma[2,1] ~ dnorm(mean.int[2],.001)

gamma[3,1] ~ dnorm(mean.int[3],.001)

gamma[1,2] ~ dnorm(mean.slp[1],.001)

gamma[2,2] ~ dnorm(mean.slp[2],.001)

gamma[3,2] ~ dnorm(mean.slp[3],.001)

mean.int[1] ~ dnorm(0,.001)

mean.int[2] ~ dnorm(0,.001)

mean.int[3] ~ dnorm(0,.001)

mean.slp[1] ~ dnorm(0,.001)

mean.slp[2] ~ dnorm(0,.001)

mean.slp[3] ~ dnorm(0,.001)

# prior for the LCDM

for(i in 1:I) {

lamda0[i] ~ dnorm(-1.096, .25)

# main effects

lamda1[i] ~ dnorm(0, .25) T(0, )

lamda2[i] ~ dnorm(0, .25) T(0, )

lamda3[i] ~ dnorm(0, .25) T(0, )

# interaction effects

lamda12[i] ~ dnorm(0, .25) T(0, )

lamda13[i] ~ dnorm(0, .25) T(0, )

lamda23[i] ~ dnorm(0, .25) T(0, )

}}

# Supplementary MCMC analyses information

Table 4.1 presents the information for all MCMC analyses across all conditions. To reduce the autocorrelation and save the memory, each chain was thinned by 1. For all replications, only last 5000 iterations were kept.

Table 4.1. Information for MCMC Analyses

| N | MO | Number of Chains | Chain Length | Burn-in |
| --- | --- | --- | --- | --- |
| 100 | 3 | 2 | 25000 | 20000 |
| 100 | 5 | 3 | 30000 | 25000 |
| 200 | 3 | 3 | 30000 | 25000 |
| 200 | 5 | 4 | 45000 | 40000 |
| 300 | 3 | 4 | 45000 | 40000 |
| 300 | 5 | 5 | 55000 | 50000 |

Note. N represents the sample size; MO represents the number of measurement occasions.

Regarding priors, non-informative priors were used for the LCDM item parameters, growth factor parameters. However, to improve the growth model convergence, the true variance-covariance matrix parameters were used as the prior for the estimated variance-covariance matrix parameters.
